# In Search of Alternative Antibiotic Drugs: Quorum-Quenching Activity in Sponges and their Bacterial Isolates

**DOI:** 10.3389/fmicb.2016.00416

**Published:** 2016-04-05

**Authors:** Kumar Saurav, Rinat Bar-Shalom, Markus Haber, Ilia Burgsdorf, Giorgia Oliviero, Valeria Costantino, David Morgenstern, Laura Steindler

**Affiliations:** ^1^Department of Marine Biology, Leon H. Charney School of Marine Sciences, University of HaifaHaifa, Israel; ^2^The Blue Chemistry Lab Group, Department of Pharmacy, Università degli Studi di Napoli Federico IINapoli, Italy; ^3^Bioinformatics Service Unit, University of HaifaHaifa, Israel

**Keywords:** anti-virulence, quorum sensing, porifera, biofilm inhibition, pyocyanin, *Pseudomonas aeruginosa*

## Abstract

Owing to the extensive development of drug resistance in pathogens against the available antibiotic arsenal, antimicrobial resistance is now an emerging major threat to public healthcare. Anti-virulence drugs are a new type of therapeutic agent aiming at virulence factors rather than killing the pathogen, thus providing less selective pressure for evolution of resistance. One promising example of this therapeutic concept targets bacterial quorum sensing (QS), because QS controls many virulence factors responsible for bacterial infections. Marine sponges and their associated bacteria are considered a still untapped source for unique chemical leads with a wide range of biological activities. In the present study, we screened extracts of 14 sponge species collected from the Red and Mediterranean Sea for their quorum-quenching (QQ) potential. Half of the species showed QQ activity in at least 2 out of 3 replicates. Six out of the 14 species were selected for bacteria isolation, to test for QQ activity also in isolates, which, once cultured, represent an unlimited source of compounds. We show that ≈20% of the isolates showed QQ activity based on a *Chromobacterium violaceum* CV026 screen, and that the presence or absence of QQ activity in a sponge extract did not correlate with the abundance of isolates with the same activity from the same sponge species. This can be explained by the unknown source of QQ compounds in sponge-holobionts (host or symbionts), and further by the possible non-symbiotic nature of bacteria isolated from sponges. The potential symbiotic nature of the isolates showing QQ activity was tested according to the distribution and abundance of taxonomically close bacterial Operational Taxonomic Units (OTUs) in a dataset including 97 sponge species and 178 environmental samples (i.e., seawater, freshwater, and marine sediments). Most isolates were found not to be enriched in sponges and may simply have been trapped in the filtration channels of the sponge at the time of collection. Our results highlight potential for QQ-bioactive lead molecules for anti-virulence therapy both from sponges and the bacteria isolated thereof, independently on the symbiotic nature of the latter.

## Introduction

Overuse of antibiotics is one of the factors involved in emergence of drug resistant pathogens. The discovery of novel drugs is required to solve this emergent problem. The understanding of how inter-cellular microbial communication is involved in bacterial pathogenesis has revealed potential for alternative strategies to treat bacteria-mediated diseases (Rasko and Sperandio, 2010; LaSarre and Federle, 2013). Quorum sensing (QS) is a type of bacterial communication system that relies on secreted signaling molecules which regulate coordinated responses across a bacterial population (Lazdunski et al., 2004). In many cases, the responses elicited by QS signals contribute directly to pathogenesis through the synchronized production of virulence determinants, such as toxins and proteases (Rutherford and Bassler, 2012). It has been theorized that, if the signal communication that coordinates these pathogenic behaviors was blocked, bacteria would lose their ability to mount an organized assault on the host and thus their ability to form organized community structures with antibiotic resistance would be compromised (Cegelski et al., 2008). QS inhibitor compounds inactivate QS by different quorum quenching (QQ) mechanisms including enzymatic inactivation of the signal molecule (Dong et al., 2000, 2001), inhibition of signal biosynthesis (Hoang and Schweizer, 1999) and inhibition of signal detection (Yang et al., 2009; Ding et al., 2011). Although QS and QQ processes were both first discovered in marine organisms, information on QQ processes in the marine environment is lagging behind compared to the studies describing the wide presence of QQ activity in soil, plants and other terrestrial samples (e.g., Dong et al., 2000; Romero et al., 2011).

The majority of modern drugs either belongs to natural products or originate from them (Li and Vederas, 2009), with marine sponges being one of the most prolific sources of chemical diverse bioactive natural products (Blunt et al., 2015). These compounds have been studied in respect to chemical ecology and provide the sponge with defense against predation, fouling, and pathogen attack (e.g., Chu et al., 2013; Rohde et al., 2015). From a biotechnology perspective, these compounds can have a broad spectrum of action comprising anti-inflammatory, anticancer, and immune-modulating activities (e.g., Costantino et al., 2008, 2009; Mayer et al., 2010; Teta et al., 2013; Blunt et al., 2015; Kaufman et al., 2015). In this study we were interested in novel antimicrobials that are based on the QQ mechanism of action. We focused on sponges based on previous studies that demonstrated the potential of sponges as a promising source for QQ compounds (Skindersoe et al., 2008; Dobretsov et al., 2011; Pejin et al., 2014; Mai et al., 2015). The discovery and sustainable production of bioactive chemical compounds from sponges requires large amounts of tissue to be processed, which is often limited by ecological concerns (the finite availability of sponges in the sea). A growing body of evidence indicates that many of these chemical compounds are produced by the sponge-associated microbiome, which includes bacteria, archaea, fungi, and algae (Thomas et al., 2010). The isolation of bacteria responsible for production of metabolites of interest would circumvent the supply problem.

In the present study we screened 14 sponge species collected from the Red Sea and the Mediterranean Sea for QQ potential using reporter strain QSIS1 and *Chromobacterium violaceum* CV026. Eighty-six isolates deriving from six selected sponge species were further evaluated for QQ activity using *C. violaceum* CV026 and *Agrobacterium tumefaciens* NT1 (pZLR4) biosensors. Based on the data obtained from the QQ screening, extract from 17 isolates were screened further for inhibition of virulence factors. To assess whether QQ-active isolates are of likely symbiotic nature, and thus may be considered potential source of metabolites found in the sponge from which they were isolated, we determined the distribution and abundance of these 17 isolates in 97 sponge species vs. 178 environmental samples (including seawater, freshwater and marine sediments). Finally, out of the 17 QQ active isolates, four showing highest anti-virulence potential were used for metabolic profiling in view of future identification of the active molecules.

## Materials and methods

### Sponge sampling

A few cubic centimeters of sponge tissue were collected by scuba diving at 5–30 m depth from the Mediterranean (Achziv nature marine reserve) and Red Sea (Eilat, Israel). Sponges were identified morphologically following the Systema Porifera classification system (Hooper and Soest, 2002). For each sponge, samples were preserved in three different ways: (i) samples were placed individually in ziploc® plastic bags and then carried to the laboratory for bacterial isolation; (ii) samples were preserved in 90% ethanol as vouchers for DNA-based taxonomic identification (deposited in the Marine Microbiology Laboratory, Department of Marine Biology, University of Haifa, Israel); and (iii) samples were immediately frozen in liquid nitrogen upon surfacing from the dives, the frozen tissues were freeze dried using a lyophilizer, and the dried tissues were utilized for chemical extractions. Samples were collected in compliance with the 40246/2014 permit from the Israel Nature and National Parks Protection Authority.

### Preparation of sponge extracts and screening for QQ activity

Lyophilized sponge tissue was macerated and extracted thrice with choloroform, chlorofom:methanol (1:1) and methanol with sonication and then filtered. All filtrates were combined and the solvents were evaporated under vacuum using a rotary vacuum evaporator (Heidolph, Germany). Extract stocks were prepared by dissolving with methanol to give a final concentration of 16 mg/mL. QQ screening of sponge extracts were performed following previously established protocols using the QSIS1 system (Rasmussen et al., 2005a) and the *C. violaceum* CV026 assay (McClean et al., 1997).

### Isolation of heterotrophic bacteria

Samples collected in ziploc® plastic bags for bacterial isolation were washed with autoclaved calcium-magnesium free artificial sea water (ASW) to remove the loosely bound bacterial cells as well as debris. Approximately, 1 cm^3^ of the sponge tissue was cut with a sterile scalpel and immediately transferred to 9 mL of sterile ASW and grounded using mortar and pestle. Several dilutions (10^−1^ to 10^−5^) were prepared and 100 μL of each spread on different solid media. Culturing media used for the isolation of marine bacteria included: Marine agar (MA–Difco), International *Streptomyces* Project (ISP) 1 and 2, Starch Casein agar (SCA), Kuster's agar (KA) (Kumar and Kannabiran, 2010), M1 medium (M1) (Abdelmohsen et al., 2010), Casamino acid agar (CAA) (Webster et al., 2001), Luria-Bertani agar (LA-HiMedia), and Nutrient agar (NA-HiMedia) were prepared with 0.2 μm filtered natural seawater and 3 or 6% sea salt. All solid media contained Difco Bacto agar (20 g/L). Plates were incubated at 28°C and observed for growth after 2–4 weeks of incubation. Every 4 days, colonies were chosen according to their morphological characteristics and then transferred to slant culture to preserve at 4°C as well as to 20% (v/v) glycerol stock at −80°C.

### Phylogenetic analysis of isolated strains

Molecular taxonomic characterization was performed using 16S rRNA sequencing. Isolation of DNA (Rainey et al., 1996), PCR primers and PCR conditions were adopted from previous studies (Jiang et al., 2006). Briefly, part of the 16S rRNA gene was amplified using MyTaq™ Red Mix (Bioline) and the primers 27F (5′-AGAGTTTGATCMTGGCTCAG-3′) and 1492R (5′-CGGTTACCTTGTTACGACTT-3′) (Sigma-Aldrich, Germany). PCR reactions were performed in a final volume of 50 μL in a Thermal Cycler (Bioer Technology), according to the following profile: 4 min at 94°C and 35 cycles of 30 s at 94°C, 30 s at 55°C, and 90 s at 72°C followed by 5 min at 72°C. The PCR product was purified and sequenced at Hylabs (Israel) using the forward primer 27F. Some strains were sequenced in addition with the reverse primer 1492R. In these cases, the two sequences were merged to give a near full length 16S rRNA gene sequence. The sequences were compared with those available in Genbank using BLASTn (Altschul et al., 1997). For the phylogenetic analysis, sequences of the closest described type strains were obtained from EzTaxon (Kim et al., 2012). Sequences of isolates and type strains were aligned using the SINA aligner verison 1.2.11 (Pruesse et al., 2012) and the alignment was manually improved. The final alignment contained 144 sequences and 693 positions.

A maximum likelihood phylogenetic tree based on the alignment was constructed using Mega 6.06 (Tamura et al., 2013). The Kimura-two-parameter model with Gamma distributed rate variation among sites and a proportion of invariant sites (+G +I) was the best substitution model for the data according to the Bayesian information criterion (BIC) of the model test implemented in MEGA6.06. The initial tree for the heuristic search with the Nearest Neighbor Interchange (NNI) algorithm was a Neighbor-Joining tree base on pairwise distances using the maximum composite likelihood approach. Validation of reproducibility of the branching patterns was made by bootstrap based on 100 re-samplings.

### Bacterial extract preparation and screening

Isolates were grown in 50 mL of AM3 production medium (Bacterial peptone 15 g/L, soybean powder 5 g/L, soluble starch 15 g/L, glycerol 15 g/L, CaCO_3_ 2 g/L, and Sea salt 3%, adjusted to pH 7.2) in a 250 mL Erlenmeyer flask and incubated for 7 days in an incubator shaker (200 rpm) at 28°C. After incubation, equal volume of butanone was added to each culture flask and then sonicated for 30 min. The organic phase was separated and concentrated in a rotary vacuum to obtain the crude extract. Dried crude extracts were used to prepare stock solutions using methanol to obtain the final concentration of 16 mg/mL. QQ activity was tested with two biosensors *C. violaceum* CV026 following a pre-established protocol (McClean et al., 1997) and with an adaptation of the thin layer chromatography (TLC) overlay technique using *A. tumefaciens* NT1 (pZLR4) (Farrand et al., 2002; Steindler and Venturi, 2007). In brief, *A. tumefaciens* NT1 (pZLR4) was cultured in AB broth medium [containing 6% (w/v) K_2_HPO_4_, 2% (w/v) KH_2_PO_4_, 2% (w/v) NH_4_Cl, 0.6% (w/v) MgSO_4_·7H_2_0, 0.3% (w/v) KCl, 0.02% (w/v) CaCl_2_, and 0.005% (w/v) FeSO_4_·7H_2_O], supplemented with 30 μg/mL gentamicin and 0.7% (w/v) glucose. Aliquots (160 μg) of crude extracts were spotted on reverse-phase C18-TLC plate (RP-C18 TLC), air-dried, overlaid with AB medium supplemented with 0.7% agar, X-Gal (40 μg/mL), C10 HSL (100 nM), and the biosensor *A. tumefaciens* NT1 (pZLR4), and incubated overnight at 30°C. Extracts presenting potential QQ activity in both assays were further tested by an additional violacein quantification assay in which inhibition of violacein synthesis was monitored in parallel to growth inhibition in 96 well plates, with 0.8 mg of extract, following an established protocol (Martinelli et al., 2004). Briefly, an overnight culture of *C. violaceum* CV026, supplemented with OC6-HSL (0.125 μg/mL) was diluted with LB medium to an OD_600_ of 1.2 and 180 μL were added to each well followed by the addition of 0.8 mg (20 μL) of crude extracts. The 96-well plate was incubated at 28°C in an incubator shaker. After 16 h, 96-well plates were allowed to dry at 60°C until all medium had evaporated (around 6 h to overnight). DMSO (200 μL) was added onto each well and placed in a shaker until all the violacein was solubilized. The absorbance of each well was read at 590 nm using TriStar Multimode Microplate reader (Berthold Technologies GmbH& Co. KG, Germany). Turbidity at OD_660_ nm was used for growth control. This was done to test whether the detected QQ activity was not related to growth inhibition of *C. violaceum* CV026. Penicillic acid (0.025 mg/mL; Rasmussen et al., 2005b) and ampicillin (3 μg/mL) were used as the positive controls for violacein and growth inhibition assays respectively. Inhibition of violacein production or growth was calculated as percentage inhibition compared to the inhibition by the negative control (methanol).

### Antibacterial activity

The antibacterial activity of extracts was tested by agar well diffusion assay against three strains: *P. aeruginosa* PAO1 (PAO1)*, Bacillus subtilis* CU1050 (BS), and *Escherichia coli* GM1655 (EC) as previously described (Saurav and Kannabiran, 2012). Briefly, 50 μL from the crude extract stock (16 mg/mL) were added in each well and the plates were incubated at 37°C for 24 h, after which activity was evidenced by the presence of a zone of inhibition surrounding the well. Each test was repeated three times and the antibacterial activity was expressed as the mean diameter of the inhibition zones (mm) produced by the tested extracts. Streptomycin was used as positive control for PAO1, and BS and ampicillin for EC. Methanol (solvent, in which the extracts were re-suspended) was used as a negative control. Further, minimum inhibitory concentration (MIC) was determined by broth 2-fold micro dilution method (CLSI M100-S20; Clinical and Laboratory Standards Institute, 2007) for 17 QQ positive isolate extracts. Briefly, the crude extracts were serially diluted (2000-4 μg/mL) using DMSO in Muller- Hinton broth. The inoculum [≈5 × 10^5^ CFU/mL (final concentration)] was prepared from an overnight culture and was added to each well containing the extract. After incubating 96-well flat bottomed plates aerobically at 37.8°C for 24 h, the OD was measured using a spectrophotometer (600 nm) to determine MIC values. Negative (Culture + DMSO) and positive controls (Ampicillin) were also included.

### Inhibition of virulence factor production-pyocyanin and protease

The assays were performed on the 17 isolates that showed QQ activity based on *C. violaceum* CV026 test. Inhibitory activity of pyocyanin production by *Pseudomonas aeruginosa* PAO1 was performed as described earlier (Pejin et al., 2014). Briefly, an overnight culture of *P. aeruginosa* PAO1 was diluted with LB medium to an OD_600_ of 0.2 and 4.5 mL were transferred to a 20 mL test tube. The diluted cultures were supplemented with 250 μL of test-extracts. Methanol and penicillic acid were used as negative and positive controls, respectively. After overnight incubation at 37°C and 200 rpm, 3 mL of chloroform were added to each test tube and mixed vigorously. The organic layer was collected by centrifugation (2000 × *g*) and transferred to a fresh tube. One milliliter of 0.2 M hydrochloric acid was added to the organic layer and the absorbance was measured at 520 nm. The experiments were performed in triplicate. Pyocyanin concentration (μg/mL) was calculated as:

(1) $$\begin{array}{l} P=\left(OD\times 17.072\right)1.5 \end{array}$$

where *OD* is optical density value obtained at 520 nm, 17.072 is the extinction coefficient to obtain the value in μg/mL, and 1.5 is the dilution factor (3 mL from initial 4.5 mL of chloroform were used; El-Fouly et al., 2015).

For assessing protease activity assay, 2 mL of LB broth were supplemented with extracts at non-inhibitory concentration (NIC) and with 1% (20 μL) of PAO1 O/N culture (OD_600_ of 0.4). DMSO and penicillic acid were used as negative and positive controls, respectively. After 18 h of incubation at 37°C, the protease activity was determined by a skim milk plate assay (Chu et al., 2013). Briefly, 100 μL of cell free supernatant of treated (extracts and penicillic acid) and untreated (negative control) PAO1 was loaded in each well containing Muller Hinton Agar with 2% skim milk and was incubated at 37°C for 24 h. Protease activity was evidenced by the presence of a zone of casein hydrolysis surrounding the well. Each test was performed in duplicates and its activity was expressed as the mean diameter of the hydrolysis zones (mm) produced by the tested extracts.

### Biofilm inhibition assay

Inhibitory effects of extracts on biofilm formation by *P. aeruginosa* PAO1 (PAO1)*, Bacillus subtilis* (BS), and *Escherichia coli* (EC) were tested by static microtiter 96 well plate assays as previously described (Sankar Ganesh and Rai Vittal, 2015). Briefly, 100 μL of LB medium were inoculated with an equal volume (100 μL) of an overnight culture (OD_600_ of 0.1) of each strain (PAO1, BS, and EC). 0.8 mg of test-extracts was added to each well. To facilitate biofilm formation, cells were incubated statically for 18 h at 37°C. Loosely bound bacteria and medium were discarded, and the plates were air-dried for 15 min and stained with 100 μL of 1% (v/v) crystal violet for 45 min. After discarding the stain, the stained biofilms were washed with distilled water and the bound stain was extracted by the addition of 200 μL of ethanol (95%, v/v). The resulting solution (200 μL) was transferred to a clean microtiter well plate to record the absorbance at 590 nm. Experiments were performed in triplicate. Streptomycin (32 μg/mL) was used as positive control for PAO1 and BS, whereas penicillic acid (8 μg/mL) was used for EC. Methanol (solvent in which the extracts were re-suspended) was used as a negative control.

### Distribution and abundance of QQ active isolates in sponge vs. environmental samples

To determine distribution and abundance of bacteria with QQ activity in sponge vs. environmental samples (seawater, marine sediments, and fresh water), we used the Sponge Microbiome Project dataset (SMP), a recently published large dataset of 16S rRNA amplicon sequencing data deriving from sponge species and environmental samples, and part of the Earth Microbiome Project (EMP, www.earthmicrobiome.org). Samples from the SMP project were taken and processed according to standard operating procedures to ensure maximum comparability. Each sponge species was sampled at least three times and samples were collected using sterile equipment. Sample processing, sequencing and core amplicon data analysis were performed by the EMP project and all metadata was made public through the data portal (www.microbio.me/emp) (Gilbert et al., 2014). The V4 region of the 16S rRNA gene was amplified using the primers 515F and 806R and sequenced using the HiSeq2500 platform (Illumina) (Caporaso et al., 2011). Sequencing data are publicly available through the Qiita website (http://qiita.ucsd.edu/) under Project ID 1740.

Illumina reads were processed in mothur v.1.31.2 (Schloss et al., 2009). Firstly, quality-filtered, demultiplexed fastq sequences were trimmed according to quality (using thetrim.seqscommand: parameters qwindowaverage = 30, qwindowsize = 5, maxambig = 0, maxhomop = 8, minlength = 100). To minimize computational effort, files were reduced to non-identical sequences (unique.seqsand count.seqs). Non-redundant sequences were aligned (align.seqs: flip = t) to a trimmed reference bacterial database, SILVA 102 (Quast et al., 2013; pcr.seqs:start = 11894, end = 25319, keepdots = F), which was provided by mothur (Quast et al., 2013). Only sequences that aligned to the expected position were kept (screen.seqs:start = 1968, end = 4411; filter.seqs: vertical = T, trump =). Aligned reads were reduced to non-redundant sequences (unique.seqs). Chimeric sequences were detected using Uchime (chimera.uchime: dereplicate = t) (Edgar et al., 2011), and filtered out (remove.seqs). Pairwise distances between aligned sequences were calculated (dist.seqs: cutoff = 0.05) and were used for clustering. Prior to clustering, aligned sequences were phylogenetic classified based on the trimmed SILVA database (classify.seqs) (Wang et al., 2007). Sequences were clustered (cluster.split: fasta =, count =, taxonomy =, splitmethod = classify, taxlevel = 4, cutoff = 0.03, hard = t, method = furthest) and converted to .shared file format (make.shared: list =, count =, label = 0.03). Finally, OTU representative sequences were retrieved based on the distance among the cluster sequences (get.oturep: list =, label = 0.03, fasta =, count =, method = abundance) and were further classified based on SILVA, Greengenes, and RDP taxonomies (classify.seqs: fasta =, template =, taxonomy =, cutoff = 60; DeSantis et al., 2006; Cole et al., 2009; Quast et al., 2013). Furthermore, Fastq sequences from additional samples (*n* = 340) that were generated at a later time point were processed with the same pipeline. These sequences were integrated into the shared file using QIIME 1.8 (Caporaso et al., 2010), based on their similarity to the OTU representative sequences (parallel_pick_otus_uclust_ref.py: –similarity 0.985 –optimal_uclust). Sequences that were not similar to the OTU representative sequences were separately clustered with mothur and integrated into the previous files (.shared and taxonomy files). The integrated OTU table (.shared file) was filtered to remove low-abundance sequences (sequences less than 0.001% across the whole dataset) and chloroplasts (according to SILVA or Greengenes). Additionally, counts from seawater-like OTUs (>0.01 % across all seawater samples) were removed from sponge samples. File manipulation and processing was carried out with python scripts (http://www.python.org). A subset of this dataset was used for downstream analyses, and included only samples with at least 2 replicates (Table S1).

16S rRNA gene sequences derived from 17 QQ-active bacteria were blasted (Camacho et al., 2009) against a subset of the Sponge Microbiome dataset consisting all the samples of minimum 2 replicates and total number of reads higher than 3000, and included 937 adult sponge samples (97 sponge species), 85 sponge larvae samples, 142 seawater samples, 33 marine sediment samples, and 3 fresh water samples. The OTUs with bit score higher than 172 and sequence identity ≥98% to the sequences of our isolates were used to determine the relative abundance to closely related bacteria among different sponge species and environmental samples. Relative abundance (%) was calculated as:

(2) $$\begin{array}{l} Relative\;abundance\left(\%\right)=\frac{x}{y}\times 100 \end{array}$$

where *x* is the sum of reads in the SMP matching to all the relevant OTUs (OTUs with bit score >172 and sequence identity ≥98% to our isolates) and *y* is the total number of reads in each sample.

The graph showing the relative abundance of OTUs in sponge vs. environmental samples was generated using an aligned dot plot graph in GraphPad Prism 5.02. Data is expressed as mean ± standard error.

### Bioassay-guided fractionation and metabolic profiles of selected strains

Extracts from 4 strains were selected for metabolic profiling using LC-HRMS/MS based on the QQ and virulence activity. To ensure that the active compound elutes from the column, a bioassay guided fractionation was performed. Crude extracts of the four selected isolates were dissolved in methanol at a concentration of 25 mg/mL. A Waters spherisorb S10 ODS2 column (4.6 × 200 mm) was used for the separation and attached to the HPLC. Two solvents were used as mobile phase: Phase A, water:acetonitrile (90:10) with 0.1% formic acid, and Phase B, acetonitrile:water (90:10). The elution procedure consisted of a linear gradient from 5 to 100% of phase B over 23 min, and an isocratic profile over 5 min with 100% phase B, followed by a isocratic profile for 2.5 min with 5% of Phase A. The volume of sample injected was 40 μl of extract. A biassay guided fractionation was followed to check the successful elution of the active compound. Fractions were collected at 7 min time intervals (fraction 1: 0–7 min, fraction 2: 7.01–14 min, fraction 3: 14.01–21 min and fraction 4: 21.01–30 min). For each extract three injections were done in order to collect sufficient amount for QQ assay (well diffusion assay using *C. violaceum* CV026, described previously).

LC-HRMS/MS experiments were carried out on a maXis ESI–QTOF Instant Expertise™, coupled to a Thermo Scientific Dionex UltiMate 3000 Rapid Separation LC (RSLC) system equipped with a UV/Vis detector acquiring at 215 and 290 nm. The four selected samples were separated on the same column as used for the bioassay-guided fractionation, using the same solvents and gradients. Samples were acquired using the following parameters: MS1 and MS2 were acquired at 1 Hz. MS1 scan was limited between 50 and 1500 *m/z* and the top 3 ions were selected for fragmentation in CID (collision induced dissociation). Acquired precursor was excluded for 30 s after two MS2 events. Data was recorded and analyzed using the software Bruker compass Data Analysis 4.2.

Raw LC-MS data was initially converted to mzXML format by Mass Convert tool Proteo Wizard (ASFPHA. 2015. Available: http://proteowizard.sourceforge.net/). The converted data (mzXML format) was then imported onto XCMS Online (Tautenhahn et al., 2012; Macintyre et al., 2014). Parameters were selected to match the instrument setup (UPLC/Q-TOF pos) with slight modifications. Various parameters were set and modified for feature detection (Tautenhahn et al., 2008), retention time correction (Prince and Marcotte, 2006) alignment (Smith et al., 2006) and annotation (Kuhl et al., 2012) to obtain the best results, for example under feature detection mode, m/z tolerance deviation of 40 ppm was set and mass tolerance level for the identification step was set to 50 ppm. All results obtained are directly linked to the METLIN metabolite database such that metabolite details and MS/MS data (if available) are displayed when a database hit is selected. Further, compounds were identified with the aid of existing high resolution MS from in-house database AntiMarin (Tawfike et al., 2013) for marine secondary metabolites. Compounds with a match were further evaluated for their UV and exact mass from previous reports. Components with no match in the databases were considered as putative novel metabolites.

### Data deposition

The sequences of the isolated bacteria used in this study were deposited in GenBank (NCBI) with the serial accession numbers KU196823-KU196855, KU206727-KU206761, and KU206763-KU206780. Sequences of the SMP/EMP OTUs with 98–100% identity to the 16S rRNA gene sequences of the 17 selected isolates can be found at the Qiita website (https://qiita.ucsd.edu/) under project ID 1740.

## Results

### QQ activity in sponge extracts

QQ activity was found in half of the sponge species tested. Out of 14 marine sponge species collected, each in triplicate, the extract of seven species showed QQ activity based on the QSIS1 assay (species were considered positive if activity was detected in at least two replicates). In the CV026 assay four of the 14 sponge species showed QQ activity, all four were found QQ-active also according the QSIS1 assay. These four sponge species included two Red Sea sponges, *Suberites clavatus* and *Negombata magnifica*, and two Mediterranean Sea species, *Ircinia variabilis*, and *Sarcotragus* sp. (Table 1). *Amphimedon chloros* extract showed QQ activity in the QSIS1 assay, but due to presence of high antimicrobial activity against CV026, its QQ activity could not be confirmed via CV026 assay. The extracts of the Red Sea sponges *Diacarnus erythraenus* and *Theonella swinhoei* were active only based on the QSIS1 system.

**Table 1:** **Quorum quenching (QQ, based on reporter strain QSIS1 and ***C. violaceum*** CV026) and antimicrobial activity (AM, against ***C. violaceum*** CV026) of sponge extracts from 14 different sponge species**.

**Sponges**	**CV026**		**QSIS1**	**Origin of sponge**
	**QQ**	**AM**	**QQ**	
*Amphimedon chloros*	ND	+	+	RS
*Axinella verrucosa*	−	−	−	MS
*Chondrosia reniformis*	−	−	−	MS
*Crella cyathophora*	−	−	−	RS
*Diacarnus erythraenus*	−	−	+	RS
*Ircinia variabilis*	+	+	+	MS
*Negombata magnifica*	+	+	+	RS
*Petrosia ficiformis*	−	−	−	MS
*Phorbas* sp.	−	−	−	MS
*Pione vastifica*	−	−	−	RS
*Sarcotragus* sp.	+	+	+	MS
*Siphonochalina siphonella*	−	−	−	RS
*Suberites clavatus*	+	+	+	RS
*Theonella swinhoei*	−	−	+	RS

*A species was considered positive if at least 2 out of 3 replicates were positive for the tested activity. Abbreviation used: RS, Red Sea; MS, Mediterranean Sea; ND, Not Determined*.

### Isolation and identification of cultivable bacteria

**Figure 1:**
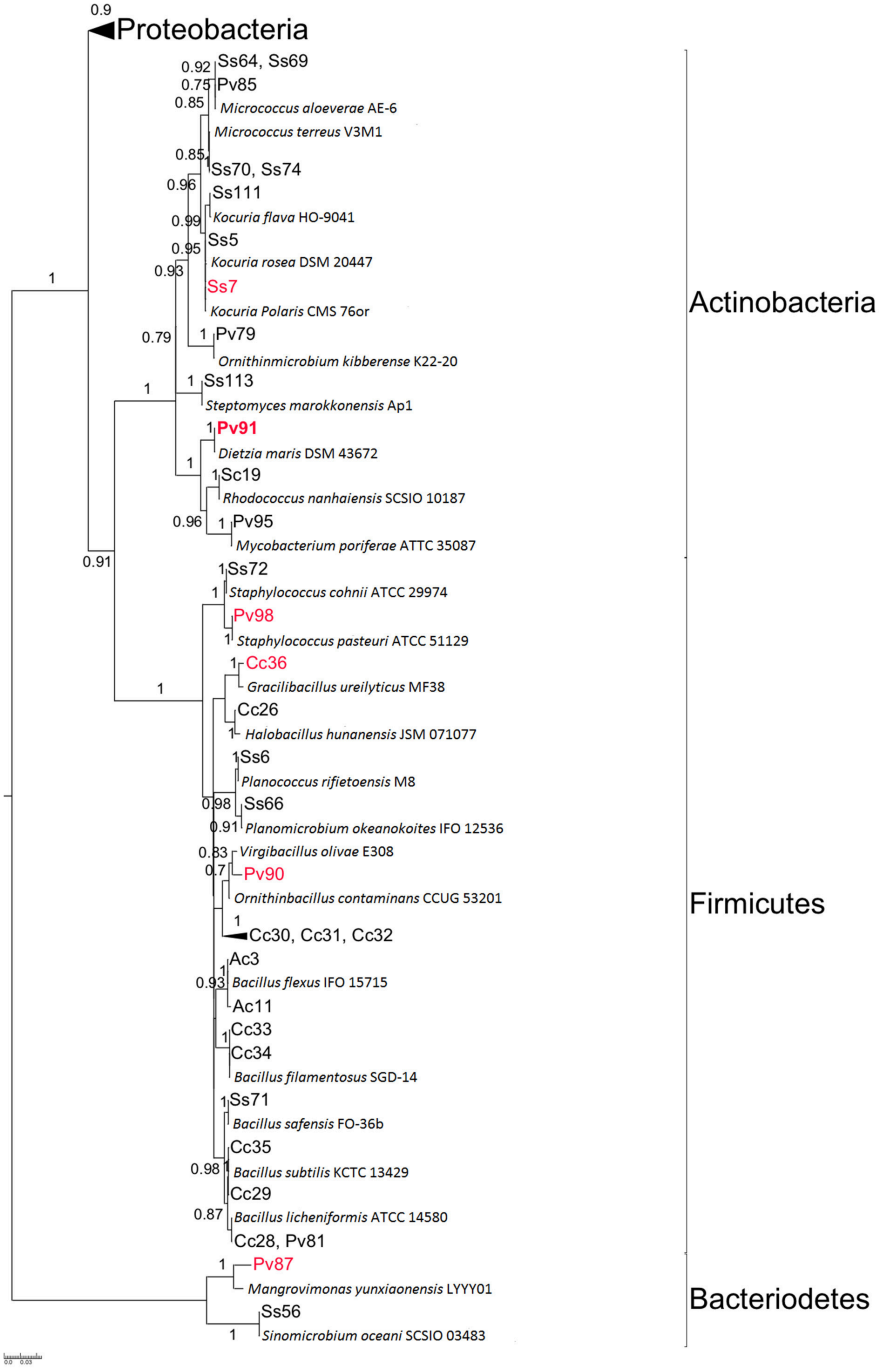
**Maximum likelihood tree of isolates and their closest EzTaxon hits**. The detailed view on the Proteobacteria is shown in Figure 2. Isolates with QQ activity are highlighted in red. Isolates in bold were used for further chemical analysis by LC-MS/MS. Bootstrap values > 70% (0.7) are shown. Strain ID provides information on the sponge source of each isolate: Ac, *Amphimedon chloros*; Cc, *Crella cyathophora*; Pv, *Pione vastifica*; Ss, *Sarcotragus* sp.; Sc, *Suberites clavatus*.

**Figure 2:**
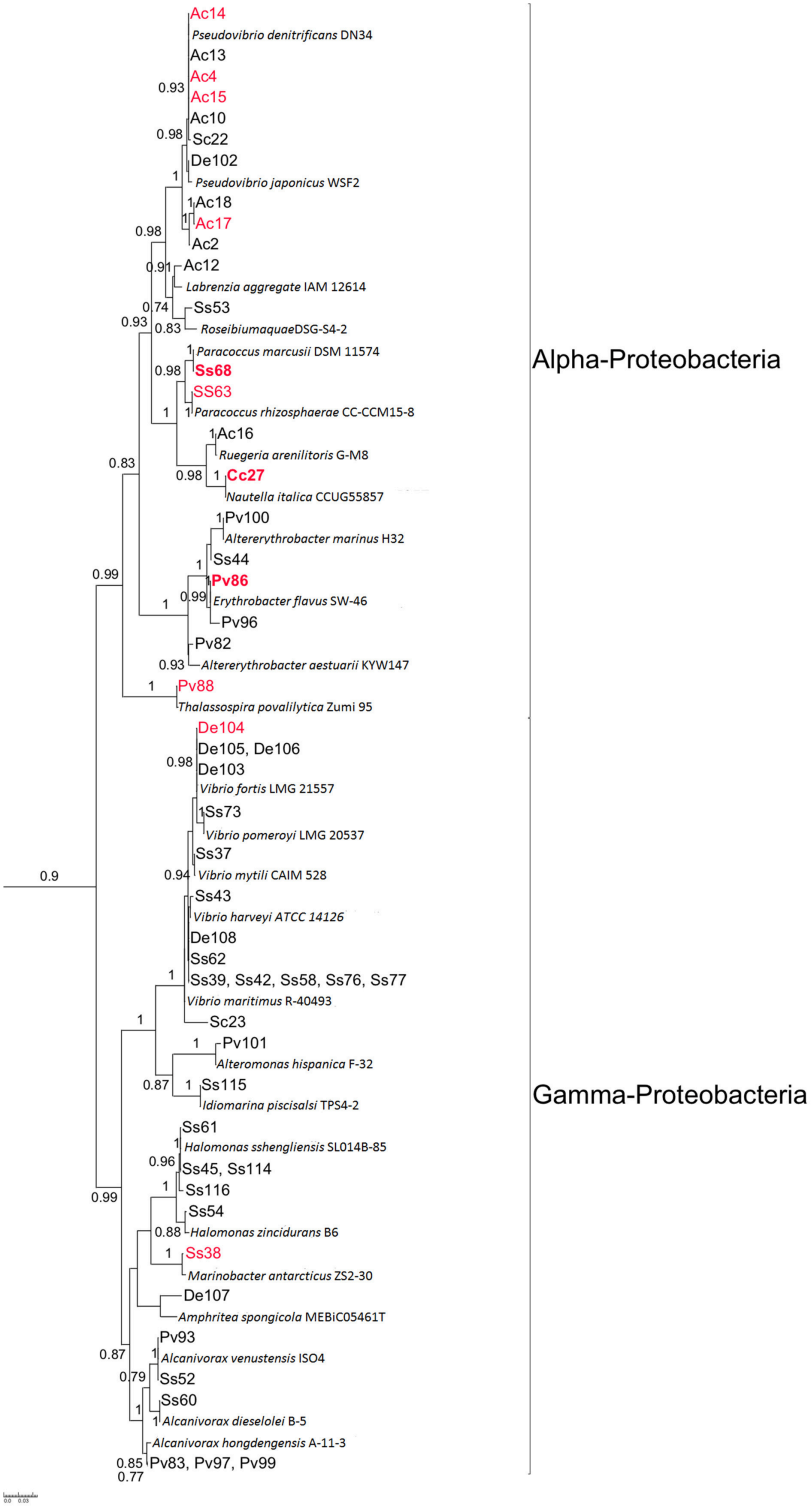
**Detailed view on Proteobacteria sub-tree from the maximum likelihood tree shown in Figure 1**. Isolates with QQ activity are highlighted in red. Isolates in bold were used for further chemical analysis by LC-MS/MS. Bootstrap values >70% (0.7) are shown. Strain ID provides information on the sponge source of each isolate: Ac, *Amphimedon chloros*; Cc, *Crella cyathophora*; Pv, *Pione vastifica*; Ss, *Sarcotragus* sp.; Sc, *Suberites clavatus*.

Six of the 14 sponge species screened for QQ activity were selected for bacteria isolation and determination of QQ potential in their extracts. These were: *A. chloros, Crella cyathophora, D. erythraenus, Pione vastifica*, and *S. clavatus* from the Red Sea and *Sarcotragus* sp. from the Mediterranean Sea. Sponge species selected included both sponges with and without QQ activity, to test whether more isolates with QQ activity could be recovered from sponges that originally showed QQ activity in their extract. All the different media used for isolation (see Materials and Methods) resulted in the isolation of bacterial strains, with Marine agar providing the highest diversity. A total of 86 representative isolates deriving from these six sponge species were selected based on morphological characteristics and RFLP analysis (data not shown) for 16S rRNA gene sequencing, and for phylogenetic analysis. Based on BLASTn and phylogenetic analysis of the partial 16S rRNA gene (>600bp), 61% of the isolated bacteria belonged to the phylum Proteobacteria, 21% to Firmicutes, 14% to Actinobateria, and 4% to Bacteroidetes (Figures 1, 2, Table 2). All the sponge species used for the isolation yielded at least one putative novel taxon with a total of 17 strains showing 16S rRNA identities ≤ 98% to previously described type strains and therefore likely representing new taxa (Table 3).

**Table 2:** **Overview of sponge isolates screened for quorum quenching activity (QQ) and antibacterial activity**.

**Isolates**	**Similarity**	**Closest related**	**Accession number**	**Class**	**Genera**	**QQ assay**	**Antimicrobial activity**		
						**CV026**	**PAO1**	**BS**	**EC**
Ac10	100	*Pseudovibrio denitrificans* UST4-50	KM196102	Alphaproteobacteria	*Pseudovibrio*	−	−	−	−
Ac11	99	*Bacillus flexus* SBANSCa10	KR186190	Bacilli	*Bacillus*	−	−	−	−
Ac12	98	*Labrenzia* sp. FGH455	JQ342690	Alphaproteobacteria	*Labrenzia*	−	−	+	−
Ac13	100	Alphaproteobacterium 5PC-32	EF657795	Alphaproteobacteria		−	−	+	−
Ac14	99	Alphaproteobacterium GUDS1146	KF282463	Alphaproteobacteria		+	−	−	−
Ac15	99	Alphaproteobacterium 5PC-32	EF657795	Alphaproteobacteria		+	−	+	−
Ac16	100	Bacterium 2D802	JF411487	Alphaproteobacteria		−	−	−	−
Ac17	96	*Pseudovibrio japonicus* U1021	AB734990	Alphaproteobacteria	*Pseudovibrio*	+	−	−	−
Ac18	97	Bacterium 1H203	JF411464	Alphaproteobacteria		−	−	−	−
Ac2	97	*Pseudovibrio japonicus* M4B33	LN812991	Alphaproteobacteria	*Pseudovibrio*	−	−	−	−
Ac3	100	*Bacillus flexus* P9	KR935237	Bacilli	*Bacillus*	−	−	−	−
Ac4	100	*Alteromonadales bacterium* GUDS1275	KF282544	Gammaproteobacteria	*Alteromonas*	+	−	−	−
Cc26	99	*Halobacillus virgiliensis* S22	AM161502	Bacilli	*Halobacillus*	−	−	−	−
Cc27	99	*Nautella* sp. ZJ2706	KP301105	Alphaproteobacteria	*Nautella*	+	−	+	−
Cc28	100	*Bacillus aerius* rawirorabr 15	LC092833	Bacilli	*Bacillus*	−	−	−	−
Cc29	100	*Bacillus subtilis* ASC4	GU227615	Bacilli	*Bacillus*	−	−	−	−
Cc30	98	*Oceanobacillus* sp. Ma-21	KF958190	Bacilli	*Oceanobacillus*	−	−	−	−
Cc31	98	*Oceanobacillus* sp. Ma-21	KF958190	Bacilli	*Oceanobacillus*	−	−	−	−
Cc32	98	*Oceanobacillus* sp. Ma-21	KF958190	Bacilli	*Oceanobacillus*	−	−	−	−
Cc33	100	*Bacillus endophyticus* HSL6	KP866218	Bacilli	*Bacillus*	−	−	−	−
Cc34	100	*Bacillus endophyticus* HSL6	KP866218	Bacilli	*Bacillus*	−	−	−	−
Cc35	100	*Bacillus subtilis* Pls8	KR709146	Bacilli	*Bacillus*	−	−	−	−
Cc36	99	*Gracilibacillus* sp. IBP-VN3	HM587330	Bacilli	*Gracilibacillus*	+	−	+	−
De102	100	*Alpha proteobacterium* 9PC-7	EF657826	Alphaproteobacteria		−	−	−	−
De103	100	*Vibrio fortis*	KM041184	Gammaproteobacteria	*Vibrio*	−	−	−	−
De104	100	*Vibrio fortis* CAIM 1769	HM584115	Gammaproteobacteria	*Vibrio*	+	−	+	−
De105	100	*Vibrio pelagius*	KM041188	Gammaproteobacteria	*Vibrio*	−	−	−	−
De106	100	*Vibrio fortis* M1C_12m_09	KM041184	Gammaproteobacteria	*Vibrio*	−	−	−	−
De107	97	*Hahellaceae bacterium* Ez249	HE818298	Gammaproteobacteria	*Hahellaceae*	−	−	−	−
De108	99	*Vibrio* sp. S2-26	KM216237	Gammaproteobacteria	*Vibrio*	−	−	−	−
Pv100	99	*Altererythrobacter marinus* H32	NR_116432	Alphaproteobacteria	*Altererythrobacter*	−	−	−	−
Pv101	100	*Alteromonas* sp. PRIM-21	KJ210054	Gammaproteobacteria	*Alteromonas*	−	−	+	−
Pv79	99	Bacterium RB26DO	KP684372	Actinobacteria		−	−	−	−
Pv81	99	*Bacillus* sp. NCIM2637	KT291161	Bacilli	*Bacillus*	−	−	−	−
Pv82	98	*Erythrobacter* sp. HME6855	HQ449708	Alphaproteobacteria	*Erythrobacter*	−	−	−	−
Pv83	98	*Alcanivorax* sp. E46	KJ364611	Gammaproteobacteria	*Alcanivorax*	−	−	−	−
Pv85	100	*Micrococcus luteus* CL10	KR780393	Actinobacteria	*Micrococcus*	−	−	−	−
Pv86	100	*Erythrobacter* sp. CUA-870	KJ732928	Alphaproteobacteria	*Erythrobacter*	+	−	−	−
Pv87	94	*Formosa* sp. CZ-BI2	JX306765	Flavobacteriia	*Mangrovimonas*	+	−	+	−
Pv88	99	Marine bacterium SE96	AY038924	Gammaproteobacteria	*Halomonadaceae*	+	−	+	−
Pv90	97	*Bacillus nitritophilus* IB-256	AJ309562	Bacilli	*Bacillus*	+	−	−	−
Pv91	100	*Dietzia maris* IHBB 9296	KR085794	Actinobacteria	*Dietzia*	+	−	+	−
Pv93	99	*Alcanivorax* sp. JXH-324	KR012312	Gammaproteobacteria	*Alcanivorax*	−	−	+	−
Pv95	100	*Mycobacterium* sp. 13-17-1	KM886164	Actinobacteria	*Mycobacterium*	−	−	+	−
Pv96	98	*Erythrobacter* sp. 32NM FL03	KM357374	Alphaproteobacteria	*Erythrobacter*	−	−	−	−
Pv97	98	*Alcanivorax* sp. 2A75	AB435642	Gammaproteobacteria	*Alcanivorax*	−	−	−	−
Pv98	100	*Staphylococcus pasteuri* SMJ33	KT036409	Bacilli	*Staphylococcus*	+	−	−	−
Pv99	99	*Alcanivorax* sp. 2A17	AB435641	Gammaproteobacteria	*Alcanivorax*	−	−	−	−
Sc19	99	*Rhodococcus nanhaiensis* SCSIO 10197	JN582176	Actinobacteria	*Rhodococcus*	−	−	+	−
Sc22	95	Alphaproteobacterium D21	DQ399723	Alphaproteobacteria		−	−	−	−
Sc23	96	*Vibrio ponticus* H078	KJ577073	Gammaproteobacteria	*Vibrio*	−	−	−	−
Ss111	99	*Kocuria* sp. MNmon19 SNSum1	KP639593	Actinobacteria	*Kocuria*	−	−	−	−
Ss113	99	*Streptomyces* sp. 1_C7_48	EF540458	Actinobacteria	*Streptomyces*	−	−	−	−
Ss114	99	*Halomonas* sp. JSM 102013	KR109186	Gammaproteobacteria	*Halomonas*	−	−	−	−
Ss115	99	*Idiomarina* sp. JL974	DQ985042	Gammaproteobacteria	*Idiomarina*	−	−	−	−
Ss116	99	*Halomonas alimentaria* HQB612	KT758546	Gammaproteobacteria	*Halomonas*	−	−	−	−
Ss37	100	*Vibrio tubiashii* 04/002-1T2	KF270470	Gammaproteobacteria	*Vibrio*	−	−	−	−
Ss38	99	*Marinobacter* sp. LV10R510-8	KF384121	Gammaproteobacteria	*Marinobacter*	+	−	−	−
Ss39	99	*Vibrio owensii* HQB622	KT758552	Gammaproteobacteria	*Vibrio*	−	−	−	−
Ss42	100	*Vibrio* sp. BL0118	KT731385	Gammaproteobacteria	*Vibrio*	−	−	−	−
Ss43	99	*Vibrio harveyi isolate* M1B39	LN812999	Gammaproteobacteria	*Vibrio*	−	−	−	−
Ss44	99	*Erythrobacter vulgaris* Nordv6	KC462903	Alphaproteobacteria	*Erythrobacter*	−	−	−	−
Ss45	99	*Halomonas* sp. JSM 102013	KR109186	Gammaproteobacteria	*Halomonas*	−	−	−	−
Ss5	100	*Kocuria rosea* 0112ALTP5	LN867187	Actinobacteria	*Kocuria*	−	−	−	−
Ss52	100	*Alcanivorax* sp. JXH-324	KR012312	Gammaproteobacteria	*Alcanivorax*	−	−	−	−
Ss53	97	*Roseibium denhamense* M2B5	LN812987	Alphaproteobacteria	*Roseibium*	−	−	−	−
Ss54	99	*Chromohalobacter* sp. Sa11	AB305300	Gammaproteobacteria	*Chromohalobacter*	−	−	−	−
Ss56	100	*Sinomicrobium oceani* AAl04,	KJ754140	Flavobacteriia	*Sinomicrobium*		−	−	−
Ss58	100	*Vibrio harveyi* ATCC 33843	CP009467	Gammaproteobacteria	*Vibrio*	−	−	−	−
Ss6	99	*Planococcus rifietoensis* CCMM B654	FR695449	Bacilli	*Planococcus*	−	−	+	−
Ss60	99	*Alcanivorax* sp. NCCP-902	AB970645	Gammaproteobacteria	*Alcanivorax*	−	−	−	−
Ss61	100	*Halomonas shengliensis* RB 40	KJ939459	Gammaproteobacteria	*Halomonas*	−	−	−	−
Ss62	99	Bacterium SCSIO13137	KJ802238	Gammaproteobacteria		−	−	−	−
Ss63	100	*Paracoccus* sp. PaH2.06a	GQ391948	Alphaproteobacteria	*Paracoccus*	+	−	−	−
Ss64	100	*Micrococcus* sp. SK57	LC068964	Actinobacteria	*Micrococcus*	−	−	−	−
Ss66	99	*Planomicrobium okeanokoites* NA-7	KC967059	Bacilli	*Planomicrobium*		−	−	−
Ss68	100	*Paracoccus* sp. JSM 2185010	KR109058	Alphaproteobacteria	*Paracoccus*	+	−	−	−
Ss69	100	*Micrococcus* sp. SK57	LC068964	Actinobacteria	*Micrococcus*	−	−	−	−
Ss7	99	*Kocuria polaris* XJB-YJ7-2	KM186611	Actinobacteria	*Kocuria*	+	−	−	−
Ss70	99	Bacterium RH70DO	KP684403	Actinobacteria		−	−	−	−
Ss71	99	*Bacillus* sp. 9RB4	KT150223	Bacilli	*Bacillus*	−	−	−	−
Ss72	100	*Staphylococcus cohnii* RCB1038	KT261250	Bacilli	*Staphylococcus*	−	−	−	−
Ss73	100	*Vibrio* sp. NB0080	KP770094	Gammaproteobacteria	*Vibrio*	−	−	−	−
Ss74	99	Bacterium RH70DO	KP684403	Actinobacteria		−	−	−	−
Ss76	99	*Vibrio* sp. PB43	KT731397	Gammaproteobacteria	*Vibrio*	−	−	−	−
Ss77	99	*Vibrio harveyi* NS131631	KR003734	Gammaproteobacteria	*Vibrio*	−	−	+	−

*Strain ID provides information on the sponge source of each isolate (Ac, Amphimedon chloros; Cc, Crella cyathophora; De, Diacarnus erythraenus; Pv, Pione vastifica; Ss, Sarcotragus sp. and Sc, Suberites clavatus). Closest 16S rRNA sequence based on % identity, and accession numbers were retrieved from NCBI database using BLASTn, and did not include uncultured/environmental sample sequences. Abbreviations used for isolates indicate the source sponge species: (Ac), Amphimedon chloros; (Cc), Crella cyathophora; (De), Diacarnus erythraenus; (Pv), Pione vastifica; (Ss), Sarcotragus sp. and (Sc), Suberites clavatus*.

**Table 3:** **List of isolates with ≤98%sequence identity to 16S rRNA genes of the closest type strains in EzTaxon**.

**Isolates**	**Closest type strain (EzTaxon)**	**Phylum**	**Similarity (%)**	**Accession no. of closest type strain**	**Base pair sequenced (bp)**
Ac2	*Pseudovibrio japonicus* WSF2	Proteobacteria	97.7	AB246748	724
Ac12	*Labrenzia aggregata* IAM 12614	Proteobacteria	96.9	AAUW01000001	615
Ac17	*Pseudovibrio japonicus* WSF2	Proteobacteria	96.5	AB246748	747
Ac18	*Pseudovibrio denitrificans* DN34	Proteobacteria	97.5	AY486423	1333
Sc23	*Vibrio maritimus* R-40493	Proteobacteria	96.8	GU929925	541
Cc26	*Halobacillus hunanensis* JSM 071077	Firmicutes	97.5	FJ425898	642
Cc30	*Ornithinibacillus contaminans* CCUG 53201	Firmicutes	97.3	FN597064	1404
Cc31	*Ornithinibacillus contaminans* CCUG 53201	Firmicutes	95.5	FN597064	680
Cc32	*Ornithinibacillus contaminans* CCUG 53201	Firmicutes	95.9	FN597064	747
Cc36	*Gracilibacillus ureilyticus* MF38	Firmicutes	97.6	EU709020	1438
Ss53	*Roseibium aquae* DSG-S4-2	Proteobacteria	96.0	KC762314	765
Ss54	*Halomonas zincidurans* B6	Proteobacteria	98.0	JQ781698	1361
Pv82	*Altererythrobacter aestuarii* KYW147	Proteobacteria	97.2	FJ997597	710
Pv87	*Mangrovimonas yunxiaonensis* LYYY01	Bacteroidetes	95.4	JQ937283	1354
Pv90	*Virgibacillus salarius* SA-Vb1	Firmicutes	96.9	AB197851	1418
Pv97	*Alcanivorax hongdengsis* A-11-3	Proteobacteria	95.3	EU438901	690
De107	*Amphritia spongicola* MEBiC05461T	Proteobacteria	92.0	GU289646	708

*Abbreviations used for isolates indicate the source sponge species: (Ac), Amphimedon chloros; (Cc), Crella cyathophora; (De), Diacarnus erythraenus; (Pv), Pione vastifica; (Ss), Sarcotragus sp. and (Sc), Suberites clavatus*.

### Quorum quenching in bacterial isolates

The butanone extracts of the fermentation broths of 86 isolates were tested for QQ activity with two bioassays (i) a well diffusion assay using biosensor *C. violaceum* CV026 and (ii) a TLC overlay assay using *A. tumefaciens* NT1 (pZLR4) as biosensor. A total of 17 crude extract (~20% of the isolates) showed QQ activity according to both biosensors used. Most isolates with QQ activity (*n* = 9) belonged to the Alphaproteobacteria, followed by Firmicutes (*n* = 3), Gammaproteobacteria (*n* = 2), Actinobacteria (*n* = 2), and Bacteroidetes (*n* = 1) (Table 2, Figures 1, 2). The most active strains (Ac4, Ac17, Cc27, Ss68, Pv86) were classified on the basis of their zone of violacein production inhibition and belonged to the genera *Alteromonas, Nautella, Paracoccus*, and *Pseudovibrio*. Activity was also confirmed by inhibition of violacein production based on microtiter plate assay monitored in parallel to growth inhibitory activity. It was observed that none of the extracts from the selected strains showed any significant inhibitory activity against *C. violaceum* CV026 except for extracts Pv88 and Pv98 with mild inhibition of 11.9 and 17.3% respectively, when compared with the negative control (Figure S1).

### Antibacterial activity

Seven of the extracts showed antibacterial activity against the Gram-positive strains *B. subtilis*, with extract Cc27 showing the largest zone of inhibition against *B. subtilis* (21 mm), none exhibited significant antagonistic activity against the two Gram-negative strains (*P. aeruginosa* PAO1 and *E. coli*; Table 1). In order to confirm the growth inhibitory activity, MIC was determined for the 17 QQ positive strains and the results were tabulated in Table S2. In accordance with well diffusion assay, none of the extract exhibited significant inhibitory activity against *P. aeruginosa* PAO1 except for the extract De103 with a MIC value of 125 μg/mL.

### Inhibition of virulence factor production

**Figure 3:**
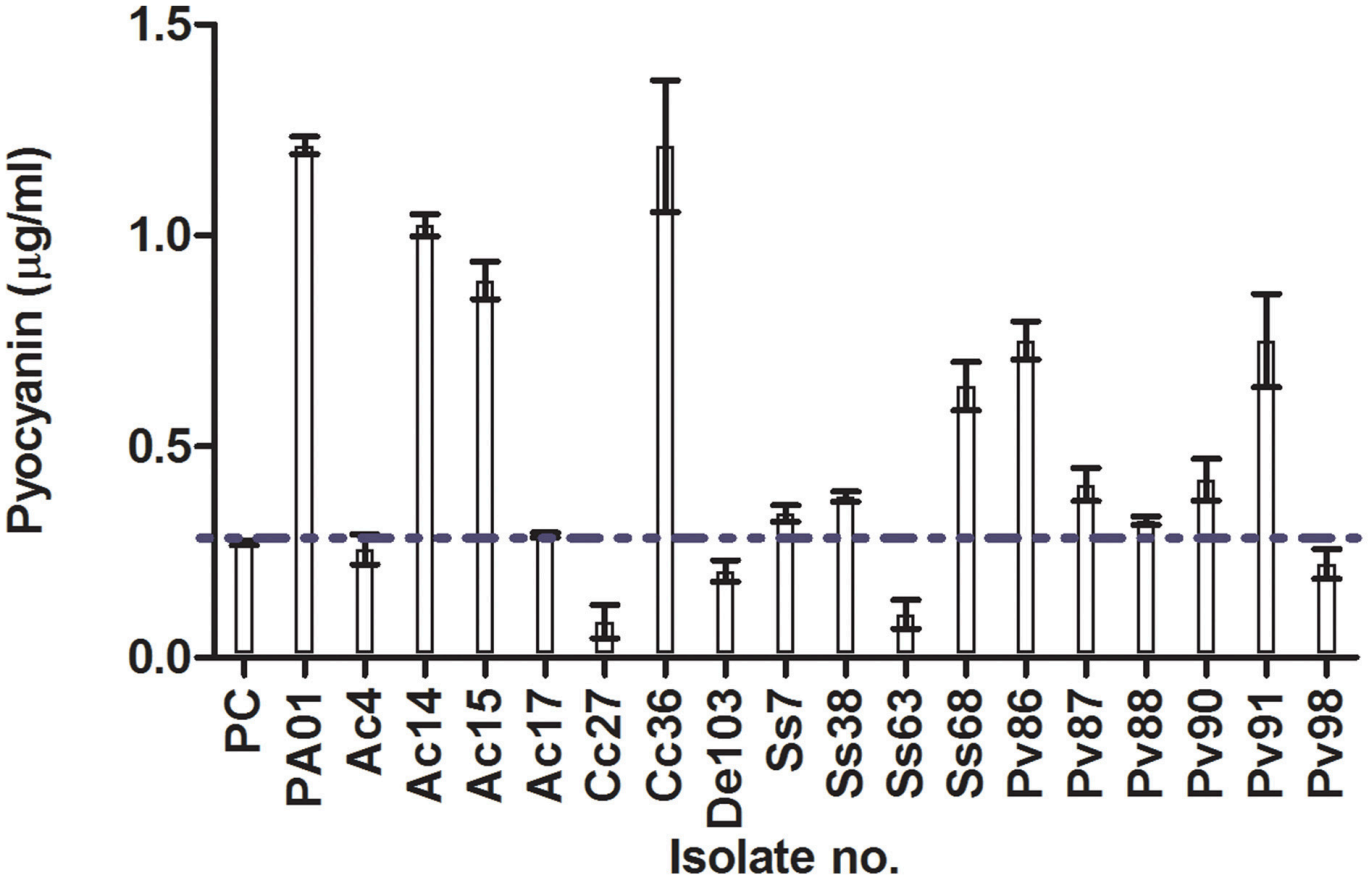
**Inhibition of pyocyanin production (μg/mL) by extracts of 17 isolated strains selected based on QQ activity**. Taxonomic information on strains can be found in Table 2. PC, Positive control (penicillic acid); PAO1, *P. aeruginosa* PAO1 grown with methanol (solvent used to dissolve extracts). Horizontal blue dotted line shows 70% of pyocyanin inhibition. Data is expressed as mean ± standard error.

All the experiments were run at NIC, to make sure that the growth of microbes is unaffected by the presence of the test extract. Extracts that were positive for QQ activity were screened for inhibition of two virulence factors that are controlled by QS in *P. aeruginosa* PAO1 (pyocyanin and protease production). Six extracts (Ac4, Ac17, Cc27, De103, Ss63, and Pv95) reduced pyocyanin production by more than 70% (Figure 3). Six extracts reduced protease production (Ac4, Ss7, Cc27, Ss68, Pv91, and De103; Table S3). Activities of De103 may be related to its growth inhibitory activity rather than QQ (see above). Protease inhibition was measured as zone of casein hydrolysis on milk agar plates. The highest zone of inhibition was observed with the extract of Ss68 (15 mm), when compared to the negative (20 mm) and positive control (16 mm).

### Biofilm inhibition

**Figure 4:**
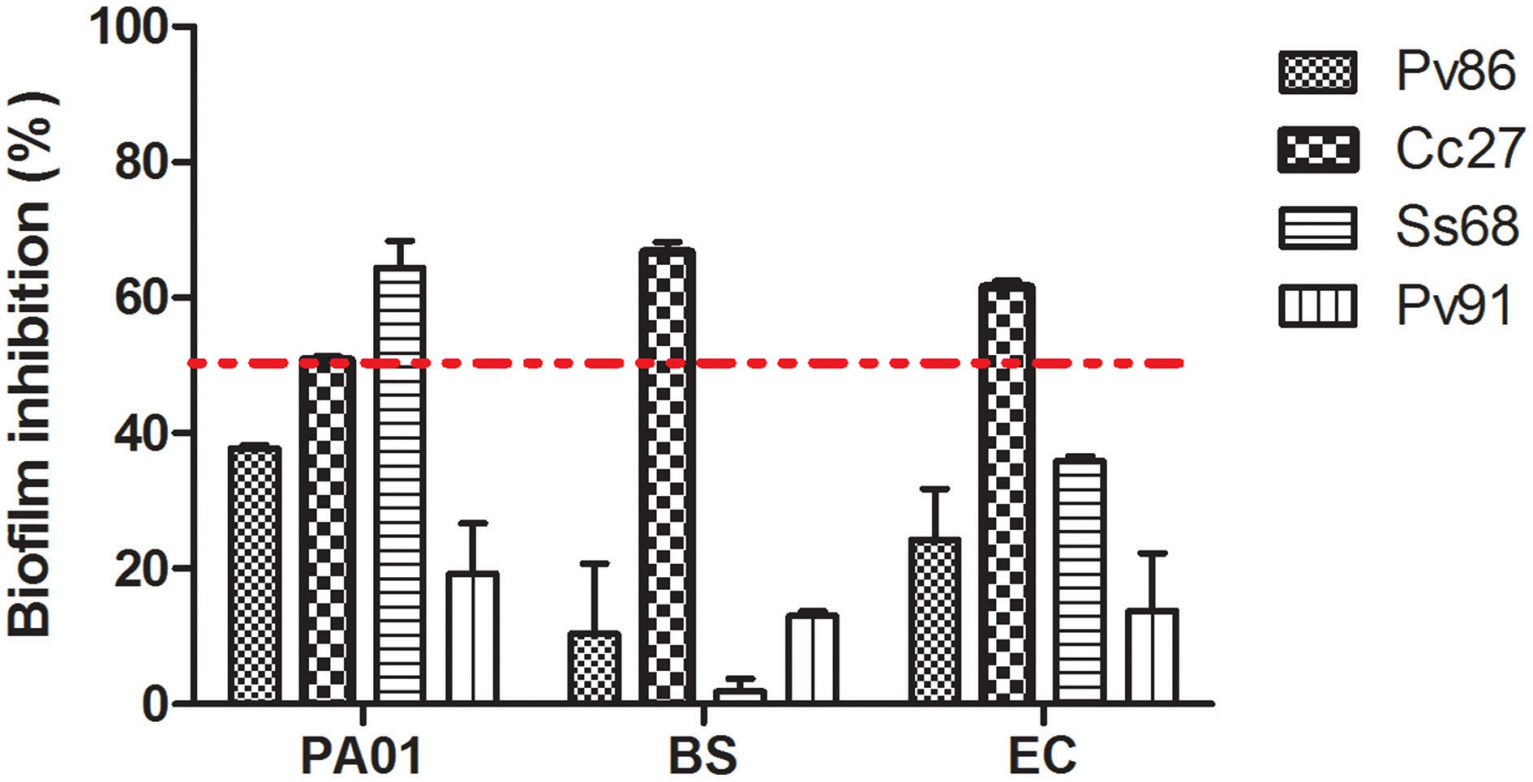
**Inhibition of biofilm formation by four selected isolates (Pv86, Cc27, Ss68, and Pv91) calculated as inhibition of biofilm formation by the isolate extract compared to that of the negative control (methanol)**. Results for biofilm inhibition in (1) *P.aeruginosa* PAO1 (PA01), (2) *Bacillus subtilis* (BS), and (3) *E. coli* (EC). Red dotted line represents 50% biofilm inhibition. Results for extracts from additional strains are shown in Supplemental Material (Figure S2). Note that some activities may have resulted from growth inhibition. Specifically extracts Ss68, Pv91, and Ss7 showed growth inhibition against BS and extract Ac4 inhibited growth of EC (see Table S2 for details on MIC). Data is expressed as mean ± standard error.

Eight extracts (Ac4, Cc27, Pv86, Pv88, Pv90, Pv91, Ss7, and Ss68) reduced biofilm formation by more than 50% in at least one of three tested bacterial strains (*P. aeruginosa* PAO1, *B. subtilis* and *E. coli*; Figure 4, Figure S2). Four strain extracts were selected for metabolic profiling based on their quorum quenching potential: three Alphaproteobacteria, Ss68 and Pv86 for showing biofilm inhibition against *P. aeruginosa* PAO1, Cc27 for showing pyocyanin inhibition and protease inhibition in *P. aeruginosa* PAO1 and also biofilm inhibition against PAO1, BS, and EC. Cc27 also showed antimicrobial potential against the Gram-positive bacteria BS, thus its biofilm inhibition of BS may have been related to antimicrobial activity. Further, the actinobacterium Pv91 was selected for its vioalcein inhibition (96 well plates QQ assay), protease inhibition in *P. aeruginosa* PAO1, and its antimicrobial activity against BS.

### Distribution and abundance of QQ active isolates in sponge vs. seawater and sediment samples

To test for a potential symbiotic nature of isolates with QQ activity, we analyzed the distribution and abundance of the QQ active isolates in the SMP dataset in sponge samples vs. seawater, freshwater and marine sediment samples. Unfortunately, this SMP dataset does not yet include the sponge species used for bacteria isolation in this study, whilst it will in the future. To account for small divergence expected in closely related symbionts found in different sponge species, we enabled some mismatches when performing the BLAST search, and included in downstream analysis any OTUs from the dataset which passed 98% identity and a bit score of 172 to our isolates. Using this criterion, out of 17 isolates tested, 15 had hits in the SMP dataset used. The OTUs are shown in Table S4.

**Figure 5:**
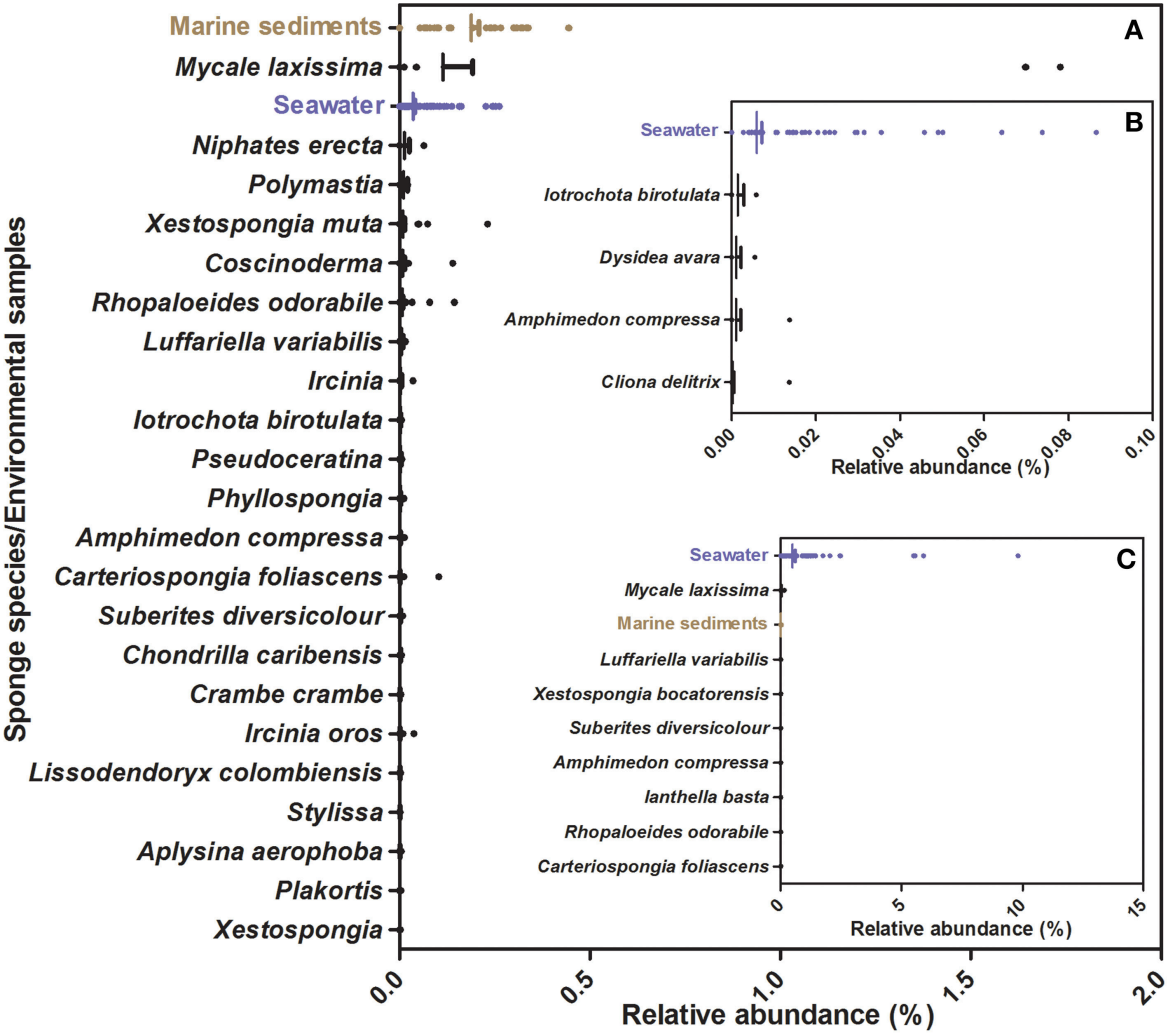
**Relative abundance of OTUs from the Sponge Microbiome Project with ≥98% identity to 16S rRNA sequences from strains isolated in this study. (A)** Information relative to OTUs closely affiliated to isolate Cc27, **(B)** Information relative to OTUs closely affiliated with isolate Pv91. **(C)** Information relative to OTUs closely affiliated with isolates Pv86. Vertical bar represents the mean, the hinge represents SEM (Standard Error of Mean), and dots represent outlier values beyond mean.

**Figure 6:**
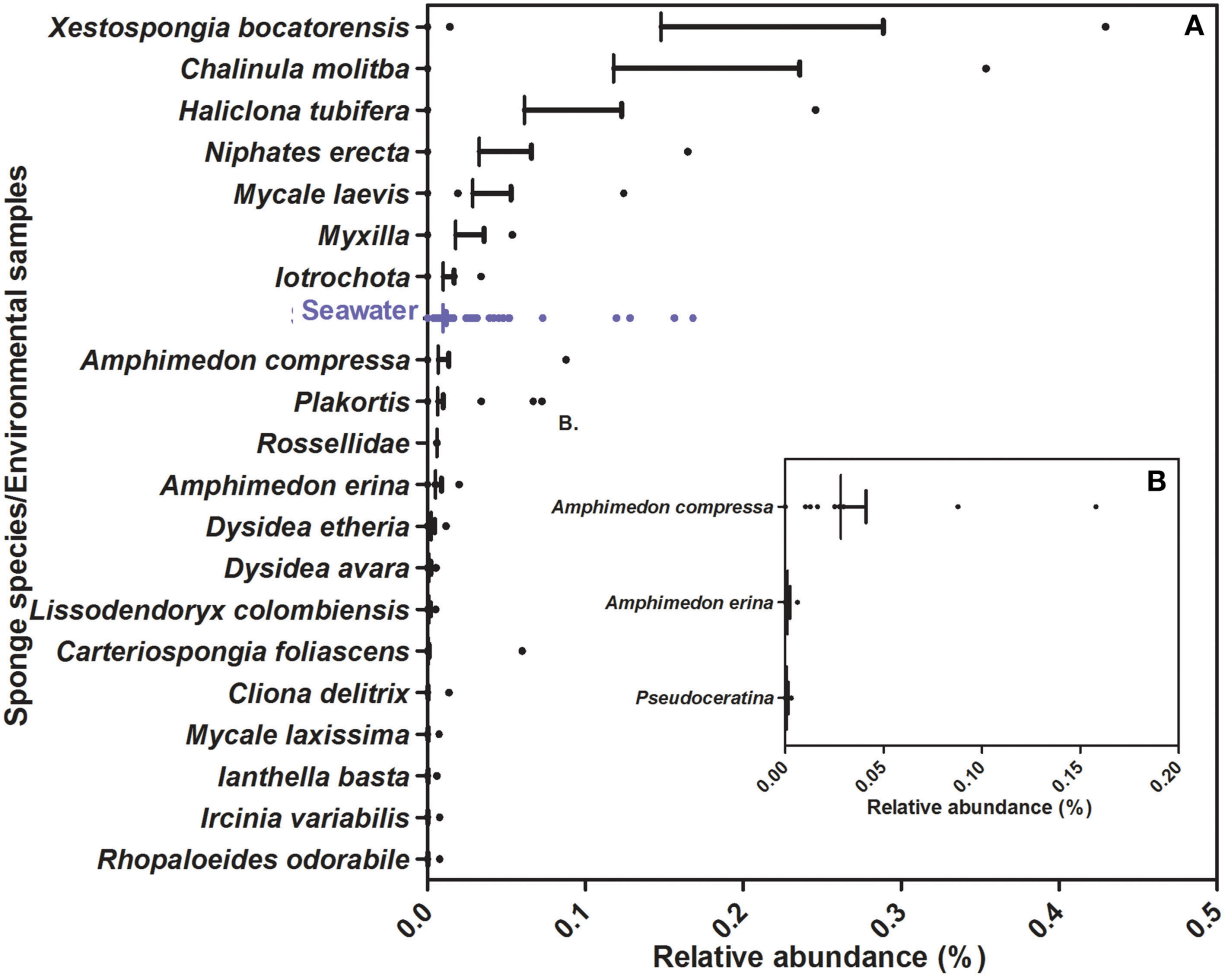
**Relative abundance of OTUs from the Sponge Microbiome Project with ≥98% identity to 16S rRNA sequences from strains isolated in this study**. **(A)** Information relative to OTUs closely affiliated to isolate Ss68, **(B)** Information relative to OTUs closely affiliated with isolate Ac17. Vertical bar represents the mean, the hinge represents SEM (Standard Error of Mean) and dots represent outlier values beyond mean.

Of these, most seems to be more common in environmental (seawater/marine sediments) than in sponge samples (e.g., Figure 5, Figures S3, S4B, S5), suggesting they may have been food-bacteria trapped in the sponge filtration system at the time of sampling. Several isolates seem to be facultative sponge-associated bacteria, found at low abundances both in sponge species and in environmental samples [e.g., Ss68 (Figure 6A), Ac4, Ac14, Ac15, (Figure S4A), Pv98 (Figure S6)]. One isolate (Ac17), from the sponge *Amphimedon chloros*, and belonging to the genus *Pseudovibrio*, showed 100% sequence identity to an OTU from the SMP dataset (OTU51717), which was present at low abundance in only three sponge species (Figure 6B), and absent from the 143 seawater and 34 sediment samples, suggesting it may be an obligatory sponge symbiont. Furthermore, 2 of 3 species in which OTU51717 was found belong to the genus *Amphimedon* (*A. compressa* and *A. erina*, Figure 6B) as for the isolation source of Ac17, *A. chloros*, suggesting a narrow host sponge range for this likely sponge-symbiont.

### Bioactive guided fractionation and metabolic profile for the four selected strains

**Figure 7:**
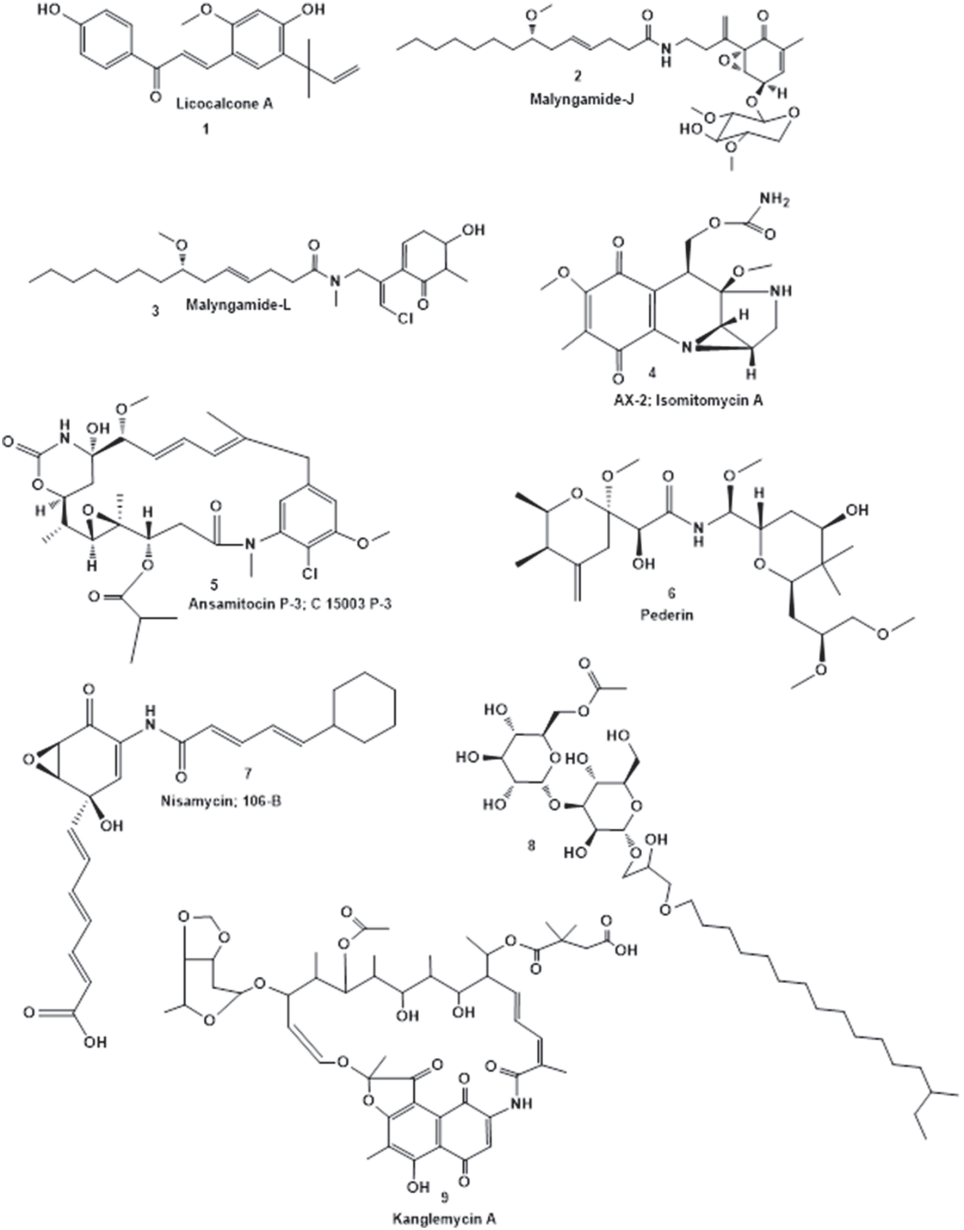
**Chemical structures of representative secondary metabolites putatively identified by LC-HRMS/MS in crude extracts of Cc27 (1-3), Ss68, Pv86 (4-6), and Pv91 (7-9) using AntiMarin database**.

Bioassay-guided fractionation resulted in the determination of active fractions on retention time scale. Fraction 2 for Cc27, fraction 3 and 4 for Ss68, fraction 4 for Pv86 and fraction 3 for Pv91 were found to be positive for QQ activity. LC-HRMS/MS profiles of the crude extracts of the four selected strains (Cc27, Ss68, Pv86, and Pv91) were further analyzed on XCMS online. Retention time drifts between the samples were compensated. An overlay of all total ion chromatograms (TICs) acquired is shown before (Figure S7A) and after (Figure S7B) retention time correction. Cloud plots were generated with 855 features (a molecular entity with a unique *m/z* and retention time, *p* ≤ 0.001) (Figure S7C), along-with location of MS/MS scans (Figure S7D). Additionally, an interactive version of the cloud plot was generated using METLIN database, in which statistics and putative identities are displayed in a pop-up window when mouse is scrolled over the circles in the plot. The shade of color is used to represent *p*-value, with brighter circles having lower *p*-values. The circles representing features with hits in the METLIN database are shown with a black filled circle (Figure S8). Selected features with their corresponding mzmed, rtmed, adduct, and METLIN MS/MS spectra are tabulated in Tables S5A–D. The most relevant chemical structures for selected metabolites were identified on the basis of their putative METLIN assignment, fragmentation pattern in mass spectra coupled with dereplication using AntiMarin database (compounds **1-9**, Figure 7).

## Discussion

Sponges are a recognized source for secondary metabolites, and reports of bioactive compounds are available for part of the sponge genera or species here analyzed: *Sarcotragus* spp. (Liu et al., 2001, 2002, 2003; Wang et al., 2008; Abed et al., 2011; Bisio et al., 2014), *Amphimedon* spp. (Watanabe et al., 1998; Ovenden et al., 1999; Matsunaga et al., 2004; Takekawa et al., 2006; Takahashi et al., 2009), *Crella cyathophora* (D'Auria et al., 1998; Ma et al., 2009), *Diacarnus* sp. (El Sayed et al., 2001; Youssef et al., 2001; Youssef, 2004; Ibrahim et al., 2008) and *Suberites* sp. (Mitova et al., 2004; Liu et al., 2009; Takahashi et al., 2011; Flemer et al., 2012; Hwang et al., 2013). However, none of these species have been previously investigated in terms of QQ activity. It is also known that space and time can affect sponge-symbiont associations and the quality and quantity of the bioactive compounds (Thomas et al., 2010) further justifying analysis of specimens of the same species when collected from new sites and different seasons. This is the first study testing for bioactive compounds in the boring clionaid sponge *Pione vastifica* and its associated bacteria. Further, this is the first study to isolate bacteria from *Suberites clavatus* and *Crella cyathophora*.

The main focus of our work was to search for inhibitors of QS as non-antibiotics based therapeutic agents against QS mediated bacterial infections. Sponges were chosen as the model animal for the search of QQ compounds for several reasons. It has been shown that bacteria inside sponges likely co-operate using QS signal molecules, the most studied being *N*-acyl homoserine lactones (Taylor et al., 2004; Mohamed et al., 2008; Gardères et al., 2012; Zan et al., 2012; Britstein et al., 2016). It can thus be expected that sponge symbionts will also compete through the quenching of QS systems, making sponges a promising source of QSI compounds. This is corroborated by a previous screen performed in the Great Barrier Reef that found QSI compounds in marine organisms, with sponges and soft corals providing the greatest numbers of QQ active extracts (Skindersoe et al., 2008). Here, we performed a screen using the same screening system (QSIS1) for sponge species collected at different geographic sites. Our results correspond to those previously reported, 46.9% of sponge species were shown to be positive for QQ in the Great Barrier Reef, and 50% were found positive in our study from the Mediterranean and the Red Sea. Our parallel screen with the CV026 system showed only 28.6% QQ-positive sponge species, however the positive sponge species were common to both QQ assays (CV026 and QSIS1), providing evidence that the CV026 is a good and fast screen method, but may result in under-representation of positive QQ samples. The discrepancy between results from the two QQ detection systems was in part related to strong antimicrobial activity of one extract against CV026 that rendered the assay impossible (extract from *Amphimedon chloros*), and likely because of intrinsic differences between the two QQ-reporter systems, QSIS1 utilizing the LuxR from *Vibrio fischeri* and *C. violaceum* CV026 using CviR.

By comparing the presence of QQ activity in extract from different sponge species and the QQ activity in extracts of bacteria isolated from the same sponge species, we could test if results from sponge extracts could be a good predictor of results from sponge isolates. For this purpose six sponge species were selected, two that had been found positive for QQ in both assays, two found positive only with QSIS1 and two negative for QQ according to both assays. The number of isolates that we identified per sponge species was variable (3–35), yet it was evident that sponges showing no QQ activity by both assay-system could provide isolates with QQ activity (e.g., *P. vastifica*, 37.5% of isolates (*n* = 16) being positive for QQ) and that sponges positive for QQ activity with both systems did not necessarily provide a higher number of isolates with QQ activity [*Sarcotragus* sp., 12.5% of the isolates (*n* = 35) being positive for QQ]. It should be noted that for testing isolate-extracts for QQ activity we only used the CV026 assay, and thus there may be an underestimation of the number of QQ active isolates. The lack of match between QQ results obtained from sponge extracts vs. extract from their bacterial isolates can be explained by the lack of knowledge on the source of the QQ compounds in complex holobiont systems, such as sponges. Both the sponge host and the isolates may produce QQ compounds and the regulation of their production is also not understood. Further, many of the isolates may not represent true sponge-associated bacteria, and thus the compounds produced by them may not be characteristic of compounds found in the sponge, from which they were isolated. The discrepancy between the natural microbial community associated to sponges as described by culture-independent studies and the cultivable microbial community has been reported before (e.g., Webster et al., 2001), hence a comparison between our isolates with QQ activity and sponge-associated bacteria as known per culture-independent techniques is informative on the likely nature (symbiotic or free-living) of our isolates. Our results on the distribution and abundance of bacteria closely related to our isolates in a wide dataset of sponge and environmental samples corroborate the likely non-symbiotic nature of most isolates that showed QQ activity. One exception being Ac17, a *Pseudovibrio* sp. that appeared present only in three sponge species and absent from all tested environmental samples (Figure 6B). Ac17 also showed 96.3% 16S rRNA identity to *Pseudovibrio* sp. POLY-S9, a strain recently isolated from an intertidal sponge of the Atlantic (Alex and Antunes, 2015). The genome of *Pseudovibrio* sp. POLY-S9 showed typical symbiotic features (e.g., ankyrin repeat proteins and tetratricopeptide repeats domain-encoding proteins) and was suggested to produce active secondary metabolites based on the presence of biosynthetic gene clusters such as polyketide synthases and nonribosomal peptide synthetases (Alex and Antunes, 2015). The potential ability of the genus *Pseudovibrio* to produce bioactive compounds has been reported before, and presence of antimicrobial activity was shown for several members of this genus (e.g., Kennedy et al., 2008; Margassery et al., 2012; Graca et al., 2013; Crowley et al., 2014; Harrington et al., 2014), whilst the potential of *Pseudovibrio* species as producers of QS-inhibitor molecules still needs further investigation. Among our 86 selected isolates 10 belonged to the genus *Pseudovibrio* and 4 showed QQ activities indicating at least a potential that warrants further investigations. Whilst most of our QQ active isolates may not represent true sponge symbionts, anti-virulence activity in their extracts and the fact that isolates represent an unlimited source of compounds, makes their non-symbiotic nature less relevant for biotechnological purposes.

Quorum sensing regulates several bacterial phenotypes essential for the successful establishment of symbiotic, pathogenic, or commensal relationships with eukaryotic hosts, including motility, exo-polysaccharide production, biofilm formation, and toxin production. *P. aeruginosa*, an opportunistic human pathogen is responsible for both acute and chronic infections. Beyond its virulence factors (e.g., pyocyanin production) its ability to form biofilm renders it more resistant to clearance by immune defense systems and by antibiotic therapy. Our screen for pyocyanin inhibition and protease activity showed that several of the extracts from QQ-active strains reduced also these QS-regulated phenotypes. Whether the observed inhibition of pyocyanin production and protease activity result from the presence of a quorum-quenching compound in the extracts used, remains to be validated. In the case of the extracts of isolates Ac4 and Cc27, the inhibition of both pyocyanin production and protease activity in *P. aeruginosa* PAO1 suggests that the mechanism of action may indeed relate to QQ compounds.

Based on the activities here described, four strains were chosen for a tentative identification of the metabolic profile of their extracts. LC-HRMS/MS analysis and comparison with existing high resolution MS records from online (METLIN) and in-house databases (AntiMarin), was used to putatively identify the presence of known and to detect the presence of unknown compounds. The extract from isolate Cc27, from *C. cyathophora*, appeared to contain a polyphenol, Licocalcone A (**1**) and two lipopeptides, Malyngamide J (**2**) and L (**3**). Licocalcone A (Fu et al., 2004) and Malyngamides (Ainslie et al., 1985) are well-known natural products, and Malyngamides have been previously isolated from marine cyanobacterium *Lyngbya majuscule* (Wu et al., 1997). Further, 8 potentially new metabolites were detected in the extract of Cc27. This isolate has 99% identity at the 16S rRNA level to *Nautella* sp. R11, a pathogen of the macroalga *Delisea pulchra*. Interestingly, this pathogen harbors a *luxR*-solo homolog (a *luxR* devoid of cognate *luxI, varR*) that is involved in colonization and virulence of the macroalga (Gardiner et al., 2015), and the macroalga is also known to produce a QS inhibitor compound (Manefield et al., 2001). LC-HRMS/MS analysis of two selected strains isolated from *Pione vastifica*, Pv86, and Pv91 showed that almost all the selected peaks in the extract of Pv86 could be putatively identified using online and in house databases (**4-6**), whereas only three of 13 selected metabolites were putatively identified from the extract of Pv91 (**7-9**). LC-HRMS/MS analysis of the extract of *Paracoccus* sp. Ss68 resulted in the putative identification of three known metabolites based on the METLIN database (METLIN ID 20991, 44851, and 18061), none of eight additional selected peaks showed matches in the AntiMarin database, suggesting this strain may produce a large number of yet unknown compounds, one of which may be the relevant one for the QQ activity observed in its extract. This study supports that QQ is a common attribute among marine sponges, and that culturable sponge associated bacteria are additional source for these compounds. Ongoing work is focused on the isolation, identification and structural elucidation of the potential QQ lead molecules produced by the selected strains.

## Author contributions

KS, LS conceived and designed the experiments; KS, RB performed the experiments; KS, MH, IB, GO, VC, DM, and LS analyzed the data; KS wrote the paper.

## Funding

This study was supported by the Bi-lateral Italy-Israel R&D grant, MOST grant#10705-3 to L. Steindler entitled: “A novel approach to fight antibiotic-resistant pathogens: acquisition of quorum sensing inhibitors from marine sponges” (by the Ministry of Science and Technology, Israel). The work was partially supported by the European Union's Seventh Framework Programme (FP7) 2007–2013 under Grant Agreement No. 311848 (Bluegenics). KS had a post-doctoral fellowship from the Israeli Council for Higher Education (VATAT) and University of Haifa, MH received a post-doctoral fellowship by the Helmsley Trust.

### Conflict of interest statement

The authors declare that the research was conducted in the absence of any commercial or financial relationships that could be construed as a potential conflict of interest.
