# Supplementary material for: In Search of Alternative Antibiotic Drugs: Quorum-Quenching Activity in Sponges and their Bacterial Isolates
Source: Front Microbiol. 2016 Apr 5;7:416. doi: 10.3389/fmicb.2016.00416 (PMC4821063; doi:10.3389/fmicb.2016.00416)
Supplement: Supplementary file 2 [file DataSheet2.pdf]

frontiers

Supplementary Material

In search of alternative antibiotic drugs: Quorum-quenching based anti virulence potential in sponges and their isolates

Kumar Saurav,[1] Rinat Bar-Shalom,[1] Markus Haber,[1] Ilia Burgsdorf, [1] Giorgia Oliviero,[2] Valeria Costantino,[2] David Morgenstern,[3] and Laura Steindler.[1*]

[1]Department of Marine Biology, Leon H. Charney School of Marine Sciences, University of Haifa, Mt. Carmel 31905, Haifa, Israel.
[2]The Blue Chemistry Lab group, Department of Pharmacy, Università degli Studi di Napoli Federico II, 80131 Napoli, Italy.

[3]Bioinformatics Service Unit, University of Haifa, Mt. Carmel 31905, Haifa, Israel.

*** Correspondence:** Corresponding Author, Laura Steindler, Department of Marine Biology, Leon H. Charney School of Marine Sciences, University of Haifa, Mt. Carmel 31905, Haifa, Israel.

Tel.: +972-48288987, lsteindler@univ.haifa.ac.il

1 Supplementary Data

Table S1. List of sponge and environmental samples used in the analysis of abundance and distribution of QQ-active isolates. Samples are part of the EMP-Sponge Microbiome dataset.
Table S2. Minimal concentration (MIC) of the butanone extracts of selected strains against *P. aeruginosa* PAO1 (PAO1), *Bacillus subtilis* CU1050 (BS), and *Escherichia coli* GM1655 (EC)
Table S3. Protease activity (mean and standard deviation of duplicates) of *P. aeruginosa* in the presence of extracts of selected strains, or controls (positive control (PC): Penicillic acid, negative control (NC): methanol).
Table S4. List of various OTUs with corresponding e-value and bit score hit from Sponge Microbiome project with ≥98% similarity against QQ-active isolates Ac4, Ac14, Ac15, Ac17, Cc27, De103, Pv86, Pv87, Pv91, Pv98, Ss38, Ss63, Ss68 and Ss7.
Table S5 A. Selected features with their corresponding mzmed, rtmed, adduct, METLIN MS/MS match and AntiMarin database match for Cc27.
Table S5 B. Selected features with their corresponding mzmed, rtmed, adduct, METLIN MS/MS match and AntiMarin database match for Ss68.
Table S5 C. Selected features with their corresponding mzmed, rtmed, adduct, METLIN MS/MS match and AntiMarin database match for Pv86.
Table S5 D. Selected features with their corresponding mzmed, rtmed, adduct, METLIN MS/MS match and AntiMarin database match for Pv91.
Figure S1 *C. violaceum* CV026 violacein and growth inhibition (%) in presence of extracts from 17 different isolates, Penicillic acid (0.025 mg/mL) and ampicillin (3 µg/mL) were used as the positive controls for violacein and growth inhibition assays respectively. Inhibition of violacein production or growth was calculated as percentage inhibition compared to the inhibition of the negative control (methanol).
Figure S2. Inhibition of biofilm formation (%) in *P. aeruginosa* PAO1, *Bacillus substilis* and *E. coli* by extracts from isolates deriving from *Crella cyathophora* (Cc), *Diacarnus erythraenus* (De), *Pione vastifica* (Pv), *Amphimedon chloros* (Ac), *Sarcotragus sp.*(Ss). Streptomycin was used as positive control (PC) for PAO1 and BS, whereas penicillic acid was used for EC. Methanol (solvent in which the extracts were re-suspended) was used as a negative control.
Figure S3. Relative abundance of OTUs from the SMP with ≥98% identity to 16S rRNA sequences from strains isolated in this study. **A.** Information relative to OTUs closely affiliated to isolate Pv87 **B**. Information relative to OTUs closely affiliated with isolate Pv88. **C**. Information relative to OTUs closely affiliated with isolate Ss38. Vertical bar represents the mean, the hinge represents SEM (standard error of mean), and dots represent outlier values beyond mean.
Figure S4. Relative abundance of OTUs from the SMP with ≥98% identity to 16S rRNA sequences from strains isolated in this study. **A.** Information relative to OTUs closely affiliated to isolates Ac4, Ac14, and Ac15 **B**. Information relative to OTUs closely affiliated with isolate Ss63. Vertical bar represents the mean, the hinge represents SEM (standard error of mean), and dots represent outlier values beyond mean.
Figure S5. Relative abundance of OTUs from the SMP with ≥98% identity to 16S rRNA sequences from strains isolated in this study. **A.** Information relative to OTUs closely affiliated to isolate De103. Vertical bar represents the mean, the hinge represents SEM (standard error of mean), and dots represent outlier values beyond mean.
Figure S6. Relative abundance of OTUs from the SMP with ≥98% identity to 16S rRNA sequences from strains isolated in this study. **A.** Information relative to OTUs closely affiliated to isolate Pv98 **B**. Information relative to OTUs closely affiliated with isolate Ss7. Vertical bar represents the mean, the hinge represents SEM (standard error of mean), and dots represent outlier values beyond mean.

Figure S7.The overlay of total ion chromatograms (A) before and (B) after retention time correction, (C) Cloud plots with 855 features with p-value of $\leq$0.001 and (D) location of MS/MS scans.

Figure S8. Interactive cloud plot analysis for the dataset containing compounds from extracts deriving from the four selected isolates (Cc27, Pv86, Pv91, and Ss68) for metabolomic profiling.

2. Supplementary Tables

Table S1. List of all sponge species and environmental sample details (including sample ID, geographical location and total number of reads) used in the analysis of distribution and abundance of OTUs closely related to QQ-active isolates in the EMP-Sponge Microbiome dataset.

Sample ID	Sponge species/ environmental sample	LATITUDE	LONGITUDE	Total number of reads
43.1020521	*Mycale laxissima*	17.95522	-67.05322	29853
46.1019503	*Mycale laxissima*	17.95522	-67.05322	25445
48.1019786	*Mycale laxissima*	17.95522	-67.05322	27172
5.11.5I.1019867	*Xestospongia muta*	26.50992	-80.03365	13593
5.11.6I.1019777	*Xestospongia muta*	26.50992	-80.03365	5840
5.11.7D.1019610	*Xestospongia muta*	26.50992	-80.03365	6321
5.11.7I.1020089	*Xestospongia muta*	26.50992	-80.03365	8025
5.11.8I.1020323	*Xestospongia muta*	26.50992	-80.03365	14712
5.11.Water.1019824	Seawater	26.509917	-80.03365	18570
5.29.4D.1020519	*Xestospongia muta*	26.16817	-80.07667	18368
5.29.5D.1019862	*Xestospongia muta*	26.16817	-80.07667	16104
5.29.7I.1020306	*Xestospongia muta*	26.16817	-80.07667	17394
50.1019597	*Mycale laxissima*	17.95522	-67.05322	36505
51.1020358	*Mycale laxissima*	17.95522	-67.05322	9769
BCD1H2O.1019563	Seawater	26.1494	-80.0971	21724
BCD2H2O.1019577	Seawater	26.1494	-80.0971	19786
BCH1H2O.1019957	Seawater	26.1494	-80.0971	23984
BCH2H2O.1020268	Seawater	26.1494	-80.0971	21346
BCH31SED.1019515	Marine sediments	26.1494	-80.0971	22063
BCH3H2O.1019849	Seawater	26.1494	-80.0971	23074
BR10A.Jan.1019548	Seawater	26.2516	-80.0623	12511
BR10A.Nov.1019707	Seawater	26.2514	-80.0621	28985
BR10C.Jul.1020245	Seawater	26.2519	-80.0632	6141
BR14A.Nov.1020043	Seawater	26.2619	-80.0851	11332
CL265.1019674	*Cliona delitrix*	24.6715	-81.0506	29294
HW14A.Jul.1019607	Seawater	26.0942	-80.1159	12546
N31.3.17.11.DC.1020439	*Amphimedon compressa*	25.842	-80.09507	16498
P10X53.1020516	*Xestospongia bocatorensis*	9.35167	-82.2595	20924
P12x11.1020349	*Erylus formosus*	9.24133	-82.17367	28627
P12x122.1020481	*Mycale laxissima*	9.35317	-82.261	6792
P12x124.1020221	*Mycale laevis*	9.35317	-82.261	10393
P12x143.1020448	*Mycale laxissima*	9.35317	-82.261	12336
P20.1020203	*Placospongia intermedia*	9.24133	-82.17367	5468
SH22SED.1019800	Marine sediments	26.1673	-80.0894	16627
SI06.11.1019752	*Xestospongia bocatorensis*	9.36068	-82.278	14908
SI06.152.1019496	*Haliclona vansoesti*	9.24133	-82.17367	6379
SI06.80.1020478	*Chalinula molitba*	9.30583	-82.17317	7636
SI06.93.1019533	*Dysidea etheria*	9.30583	-82.17317	13623
SI06.94.1019967	*Haliclona tubifera*	9.30583	-82.17317	8292

SW.245.1020115	*Halichondria panicea*	58.983333	11.276667	19712
SW.30.1020365	*Mycale lingua*	58.961667	11.3275	6384
SW.H2O.5.1019804	Seawater	58.829116	11.083156	20173
SW.H2O.6.1019920	Seawater	58.829116	11.083156	22381
SWBHMPV1.1020492	Seawater	22.100867	-73.540733	29077
SWBHMPV2.1019827	Seawater	22.100867	-73.540733	22944
SWBHMPV3.1019729	Seawater	22.100867	-73.540733	27717
SWBHPK1.1020427	Seawater	22.6045	-73.546533	19438
SWBHPK2.1020243	Seawater	22.6045	-73.546533	17443
SWBHPK3.1020109	Seawater	22.6045	-73.546533	15987
SWBHSS1.1020185	Seawater	24.04055	-74.531367	27943
SWBHSS3.1020029	Seawater	24.04055	-74.531367	28825
SWPRBV2.1020241	Seawater	17.888283	-66.998117	18341
SWPRD1.1019576	Seawater	18.391767	-67.475967	29263
Webster.3.D8.232.1020434	*Carteriospongia foliascens*	-18.68543	146.51218	27620
Webster.3.F6.254.1019814	*Carteriospongia foliascens*	-18.5996	146.49222	11686
Webster.5.E6.430.1020522	Seawater	-18.82257	147.63755	22939
AF11.7.9.1181192	*Sarcotragus fasciculatus*	41.68	2.81	23671
AW10.10.II.1181451	Seawater	41.68	2.81	14622
AW10.10.III.1181682	Seawater	41.68	2.81	17871
AW10.11.I.1181594	Seawater	41.68	2.81	13811
AW10.11.II.1181844	Seawater	41.68	2.81	16416
AW10.12.I.1182118	Seawater	41.68	2.81	16762
AW10.12.III.1181128	Seawater	41.68	2.81	9536
AW10.3.II.1181943	Seawater	41.68	2.81	17945
AW10.3.III.1181432	Seawater	41.68	2.81	13911
AW10.4.I.1181462	Seawater	41.68	2.81	22379
AW10.4.II.1181086	Seawater	41.68	2.81	22589
AW10.4.III.1182286	Seawater	41.68	2.81	23204
AW10.5.I.1181997	Seawater	41.68	2.81	18009
AW10.7.II.1181304	Seawater	41.68	2.81	13328
AW10.7.III.1182181	Seawater	41.68	2.81	17757
AW10.8.I.1181963	Seawater	41.68	2.81	16032
AW10.8.II.1181869	Seawater	41.68	2.81	14473
AW10.8.III.1182423	Seawater	41.68	2.81	19328
AW10.9.I.1181604	Seawater	41.68	2.81	17660
AW10.9.II.1181973	Seawater	41.68	2.81	16348
AW10.9.III.1181831	Seawater	41.68	2.81	21761
AW11.1.I.1181552	Seawater	41.68	2.81	27081
AW11.1.II.1181307	Seawater	41.68	2.81	14641
AW11.1.III.1182104	Seawater	41.68	2.81	10272
AW11.2.I.1181241	Seawater	41.68	2.81	17615
AW11.2.II.1181195	Seawater	41.68	2.81	21826
AW11.2.III.1181851	Seawater	41.68	2.81	11250
AW11.3.I.1181767	Seawater	41.68	2.81	21001

AW11.4.II.1181401	Seawater	41.68	2.81	13481
AW11.4.III.1182415	Seawater	41.68	2.81	17185
AW11.6.I.1182134	Seawater	41.68	2.81	13804
AW11.6.II.1182018	Seawater	41.68	2.81	6987
AW11.6.III.1182287	Seawater	41.68	2.81	15661
AW11.8.I.1181714	Seawater	41.68	2.81	21949
AW11.8.III.1182400	Seawater	41.68	2.81	20452
IF2.WI.1181794	Seawater	41.38	2.2	25384
IF2.WII.1181395	Seawater	41.38	2.2	23010
IF5.Wctr.1181399	Seawater	41.38	2.2	11374
IF5.Wnh.1182034	Seawater	41.38	2.2	15953
IO2.WI.1181409	Seawater	41.38	2.2	20788
IO2.WII.1181646	Seawater	41.38	2.2	25035
IO2.WIII.1181978	Seawater	41.38	2.2	27143
SWBH.1.1181167	Seawater	41.67	2.8	17950
SWBH.2.1182397	Seawater	41.67	2.8	21183
SWBH.3.1181292	Seawater	41.67	2.8	23568
SWSA.1.1181494	Seawater	41.67	2.8	24091
SWSA.2.1181197	Seawater	41.67	2.8	19765
SWSA.3.1181074	Seawater	41.67	2.8	20591
TV10.11.12.1181165	*Ircinia variabilis*	41.72	2.94	30905
TV10.12.7.1181211	*Ircinia variabilis*	41.72	2.94	31798
TV10.5.12.1181986	*Ircinia variabilis*	41.72	2.94	46532
TV10.5.7.1182192	*Ircinia variabilis*	41.72	2.94	42192
TV10.7.2.1181625	*Ircinia variabilis*	41.72	2.94	33116
TV10.7.7.1182251	*Ircinia variabilis*	41.72	2.94	33327
TV10.8.7.1181883	*Ircinia variabilis*	41.72	2.94	29665
TV11.1.7.1181486	*Ircinia variabilis*	41.72	2.94	30571
TV11.2.12.1181251	*Ircinia variabilis*	41.72	2.94	34977
TV11.5.12.1181838	*Ircinia variabilis*	41.72	2.94	27536
TV11.7.12.1181394	*Ircinia variabilis*	41.72	2.94	27333
TV11.8.7.1181932	*Ircinia variabilis*	41.72	2.94	30604
TW10.10.I.1181667	Seawater	41.72	2.94	27474
TW10.10.III.1181697	Seawater	41.72	2.94	15233
TW10.11.I.1181974	Seawater	41.72	2.94	13792
TW10.11.II.1181427	Seawater	41.72	2.94	19940
TW10.11.III.1182236	Seawater	41.72	2.94	23411
TW10.12.I.1182004	Seawater	41.72	2.94	20334
TW10.12.II.1181757	Seawater	41.72	2.94	9042
TW10.12.III.1182205	Seawater	41.72	2.94	7789
TW10.3.II.1181259	Seawater	41.72	2.94	25440
TW10.3.III.1181864	Seawater	41.72	2.94	24781
TW10.4.II.1182137	Seawater	41.72	2.94	18446
TW10.4.III.1182315	Seawater	41.72	2.94	20906
TW10.6.I.1181884	Seawater	41.72	2.94	16380

TW10.6.II.1182441	Seawater	41.72	2.94	20212
TW10.7.II.1182248	Seawater	41.72	2.94	13425
TW10.8.I.1181563	Seawater	41.72	2.94	6546
TW10.8.II.1182342	Seawater	41.72	2.94	16161
TW10.8.III.1182435	Seawater	41.72	2.94	18175
TW10.9.I.1181157	Seawater	41.72	2.94	17310
TW10.9.II.1181335	Seawater	41.72	2.94	21078
TW10.9.III.1182302	Seawater	41.72	2.94	26334
TW11.1.I.1181751	Seawater	41.72	2.94	12703
TW11.1.II.1181113	Seawater	41.72	2.94	10239
TW11.1.III.1181629	Seawater	41.72	2.94	14267
TW11.2.I.1181107	Seawater	41.72	2.94	18401
TW11.2.III.1181469	Seawater	41.72	2.94	17194
TW11.3.I.1181174	Seawater	41.72	2.94	22300
TW11.3.II.1181991	Seawater	41.72	2.94	16758
TW11.3.III.1182340	Seawater	41.72	2.94	13984
TW11.4.II.1181268	Seawater	41.72	2.94	12725
TW11.5.III.1182247	Seawater	41.72	2.94	17934
11.1019601	*Ircinia strobilina*	18.40198	-87.40878	6191
11.XII.84.1.014.1020284	*Axinella corrugata*	25.40333	-77.90833	35777
20.X.00.1.015.1020304	*Discodermia*	26.51255	-78.58482	14478
21.X.03.5.008.1020153	*Discodermia sp.*	26.51122	-78.58708	16948
26.IX.88.1.023.1020410	*Axinella corrugata*	24.058	-74.54217	12071
27.V.93.2.7.1020505	*Xestospongia muta*	22.055	-74.55333	3403
28.IX.88.3.001.1020352	*Axinella corrugata*	23.64667	-74.945	24068
4.VI.93.4.001.1019646	*Axinella*	22.72333	-73.88333	11930
40.1020202	*Mycale laxissima*	17.95522	-67.05322	4137
5.11.143I.1020345	*Xestospongia muta*	26.50992	-80.03365	15892
5.11.6H.1019523	*Xestospongia muta*	26.50992	-80.03365	11225
5.11.9I.1020119	*Xestospongia muta*	26.50992	-80.03365	15413
5.29.4H.1019593	*Xestospongia muta*	26.16817	-80.07667	23141
5.29.4I.1019789	*Xestospongia muta*	26.16817	-80.07667	18986
5.29.7D.1020389	*Xestospongia muta*	26.16817	-80.07667	21361
5.29.8D.1020459	*Xestospongia muta*	26.16817	-80.07667	8427
64.1020327	*Xestospongia*	17.88828	-66.99812	15915
70.1020429	*Xestospongia*	20.38228	-87.0291	24536
BCD11SED.1020055	Marine sediments	26.1494	-80.0971	15691
BCH11SED.1020281	Marine sediments	26.1494	-80.0971	20818
CL105.1020425	*Cliona delitrix*	26.14217	-80.09657	19173
CL106.1020152	*Cliona delitrix*	26.14217	-80.09657	14534
CL108.1019904	*Cliona delitrix*	26.14217	-80.09657	18469
CL109.1020300	*Cliona delitrix*	26.14217	-80.09657	12145
CL11.1019830	*Cliona delitrix*	24.54912	-81.37958	7837
CL110.1020007	*Cliona delitrix*	26.14217	-80.09657	17495
CL12.1020264	*Cliona delitrix*	24.54912	-81.37958	13414

CL13.1020533	*Cliona delitrix*	24.54912	-81.37958	11207
CL183.1019498	*Cliona delitrix*	16.80083	-88.07887	11708
CL24.1019928	*Cliona delitrix*	24.54912	-81.37958	5494
CL251.1020171	*Cliona delitrix*	26.0522	-80.09782	11188
CL299.1020078	*Cliona delitrix*	25.84217	-80.10403	25194
CL301.1019822	*Cliona delitrix*	25.84217	-80.10403	14679
CL303.1019948	*Cliona delitrix*	25.84217	-80.10403	13831
CL352.1020377	*Cliona delitrix*	24.05862	-74.54122	9111
CL353.1020282	*Cliona delitrix*	24.05862	-74.54122	44490
CL415.1019946	*Cliona delitrix*	24.60903	-82.94957	12276
CL417.1020011	*Cliona delitrix*	24.60903	-82.94957	29000
CL42.1020404	*Cliona delitrix*	9.3777	-82.3032	14376
CL420.1019933	*Cliona delitrix*	24.60903	-82.94957	15389
CL461.1020397	*Cliona delitrix*	24.94067	-80.45383	11025
Cl179.1019663	*Cliona delitrix*	16.80083	-88.07887	12650
GC.2.3.1020219	*Stylissa carteri*	20.280232	38.512573	16824
N31.5.9.11.DC.1020371	*Amphimedon compressa*	25.842	-80.09507	11396
N32.12.6.10.DC.1020311	*Amphimedon compressa*	25.842	-80.09507	10244
N32.5.9.11.DC.1019961	*Amphimedon compressa*	25.842	-80.09507	7947
N50.11.10.11.BC.1020037	*Amphimedon compressa*	26.15995	-80.0825	10147
N50.9.1.11.BC.1020044	*Amphimedon compressa*	26.15995	-80.0825	29367
P12x101.1020320	*Niphates erecta*	9.36068	-82.278	4056
P12x116.1019703	*Niphates erecta*	9.36068	-82.278	5463
P12x119.1019608	*Niphates erecta*	9.36068	-82.278	4718
P12x134.1020162	*Mycale laxissima*	9.35317	-82.261	8844
P12x144.1020206	*Mycale laxissima*	9.35317	-82.261	16389
SI06.79.1020289	*Amphimedon erina*	9.30583	-82.17317	16360
SS.11.3.1019853	*Ircinia*	-18.819402	147.649572	19184
SS.14.1.1020351	*Pseudoceratina*	13.455407	144.645146	32642
SS.14.3.1020260	*Pseudoceratina*	13.455407	144.645146	33467
SS.16.1.1019647	*Stylissa massa*	13.455407	144.645146	18085
SS.16.2.1019945	*Stylissa massa*	13.455407	144.645146	25139
SS.22.3.1020163	*Ircinia felix*	24.957076	-80.460613	27950
SWPRBV3.1019766	Seawater	17.888283	-66.998117	26540
SWPRD2.1019841	Seawater	18.391767	-67.475967	29010
SWPRD3.1020378	Seawater	18.391767	-67.475967	27249
Webster.1.D3.39.1019529	*Ianthella basta*	-18.82257	147.63755	6926
Webster.1.E1.49.1019860	*Coralliophila*	-18.82257	147.63755	8075
Webster.4.F1.343.1020468	*Coscinoderma*	-18.59297	46.48359	6291
Webster.5.B6.394.1019750	*Spheciospongia vagabunda*	-12.46725	130.829517	9968
Webster.5.E1.425.1020137	*Suberites diversicolour*	-12.4152	130.83193	11657
ZA.14.1020132	*Xestospongia*	-6.11738	39.16904	46360
ZA.17.1020226	*Cinachyrella*	-6.11738	39.16904	36528
ZA.37.1020177	*Callyspongia*	-6.149923	39.131603	17810
ZA.39.1020013	*Callyspongia*	-6.149923	39.131603	10321

ZA.44.1020227	*Cymbastela*	-6.165917	39.202641	15814
ZA.47.1020435	*Cymbastela*	-6.165917	39.202641	21596
CCBH.2.1182428	*Crambe crambe*	41.67	2.8	19719
IO5.24.1181867	*Ircinia oros*	41.38	2.2	13398
100.1019549	*Xestospongia*	17.88828	-66.99812	30984
113.1020508	*Xestospongia*	17.88828	-66.99812	30905
117.1020476	*Xestospongia*	18.39177	-67.47597	26493
3.1020215	*Mycale laxissima*	26.56295	-77.8815	18823
37.1020129	*Mycale laxissima*	17.95522	-67.05322	26856
44.102037	*Mycale laxissima*	17.95522	-67.05322	29124
45.1020225	*Mycale laxissima*	17.95522	-67.05322	6846
5.11.120H.1019638	*Xestospongia muta*	26.50992	-80.03365	22088
5.11.120I.1019774	*Xestospongia muta*	26.50992	-80.03365	30288
5.11.136H.1019859	*Xestospongia muta*	26.50992	-80.03365	17531
5.11.137H.1019552	*Xestospongia muta*	26.50992	-80.03365	17944
5.11.142D.1020350	*Xestospongia muta*	26.50992	-80.03365	9149
5.11.143D.1019573	*Xestospongia muta*	26.50992	-80.03365	14091
5.11.4I.1019698	*Xestospongia muta*	26.50992	-80.03365	17091
5.11.5H.1019892	*Xestospongia muta*	26.50992	-80.03365	17476
5.11.9H.1020261	*Xestospongia muta*	26.50992	-80.03365	12686
5.29.1H.1020444	*Xestospongia muta*	26.16817	-80.07667	21615
5.29.2H.1020009	*Xestospongia muta*	26.16817	-80.07667	17046
5.29.3H.1019531	*Xestospongia muta*	26.16817	-80.07667	15131
5.29.5H.1020252	*Xestospongia muta*	26.16817	-80.07667	15165
5.29.6I.1020436	*Xestospongia muta*	26.16817	-80.07667	19331
5.29.7H.1019812	*Xestospongia muta*	26.16817	-80.07667	14985
5.29.8H.1020181	*Xestospongia muta*	26.16817	-80.07667	12646
53.1020236	*Mycale laxissima*	17.95522	-67.05322	32088
57.1019587	*Mycale laxissima*	17.95522	-67.05322	17530
59.1019505	*Mycale laxissima*	17.95522	-67.05322	17231
60.1020005	*Mycale laxissima*	17.95522	-67.05322	23012
74.1019775	*Xestospongia*	17.88828	-66.99812	16745
81.1020014	*Xestospongia*	17.88828	-66.99812	30450
82.101958	*Xestospongia*	17.88828	-66.99812	34664
93.1020001	*Xestospongia*	17.88828	-66.99812	22851
97.1020294	*Xestospongia*	17.88828	-66.99812	44516
98.1020248	*Xestospongia*	17.88828	-66.99812	23376
ALG12.90.1019650	*Phorbas fictitius*	37.069333	-8.331138889	20469
ALG12.91.1020146	*Phorbas fictitius*	37.069333	-8.331138889	21609
ALG12.96.1019911	*Phorbas fictitius*	37.069333	-8.331138889	19310
ALG12.97.1020523	*Phorbas fictitius*	37.069333	-8.331138889	23025
BZ09x1.1019879	*Tedania ignis*	16.82983	-88.1045	34476
CL293.1019996	*Cliona delitrix*	25.84217	-80.10403	14468
CL302.1020065	*Cliona delitrix*	25.84217	-80.10403	11181
CL418.1019557	*Cliona delitrix*	24.60903	-82.94957	8830

CL52.1019677	*Cliona delitrix*	9.3777	-82.3032	13023
CL53.1020356	*Cliona delitrix*	9.3777	-82.3032	4144
GC.1.1.1019521	*Xestospongia testudinaria*	20.280232	38.512573	18100
GC.1.2.1020021	*Xestospongia testudinaria*	20.280232	38.512573	22051
GC.1.3.1020054	*Xestospongia testudinaria*	20.280232	38.512573	20609
GC.1.4.1019595	*Xestospongia testudinaria*	20.280232	38.512573	25366
GC.1.5.1020049	*Xestospongia testudinaria*	20.280232	38.512573	23147
GC.2.1.1020491	*Stylissa carteri*	20.280232	38.512573	23629
GC.2.5.1020467	*Stylissa carteri*	20.280232	38.512573	23863
N50.5.10.11.BC.1019840	*Amphimedon compressa*	26.15995	-80.0825	18222
P12x145.1020431	*Amphimedon compressa*	9.35167	-82.2595	7850
P12x147.1019555	*Amphimedon compressa*	9.35167	-82.2595	7976
P12x149.1020354	*Amphimedon compressa*	9.35167	-82.2595	9065
P12x150.1020217	*Amphimedon compressa*	9.35167	-82.2595	7917
P12x151.1020099	*Amphimedon compressa*	9.35167	-82.2595	12572
P12x54.1019994	*Amphimedon erina*	9.30583	-82.17317	9985
P12x58.1019792	*Amphimedon erina*	9.30583	-82.17317	18184
P81.1020228	*Amphimedon erina*	9.30583	-82.17317	19392
SD13SED.1020318	Marine sediments	26.1673	-80.0894	14587
SH3H2O.1020545	Seawater	26.1673	-80.0894	22690
SI06.59.1020443	*Tedania ignis*	9.33333	-82.25	13196
SI06.67.1019719	*Amphimedon erina*	9.30583	-82.17317	32650
SI06.78.1020367	*Tedania ignis*	9.30583	-82.17317	17398
SS.17.3.1020518	*Xestospongia*	13.455407	144.645146	17982
SS.20.3.1019724	*Ircinia*	-32.134084	115.766008	12225
SS.24.1.1019990	*Xestospongia muta*	24.957076	-80.460613	23738
SS.24.2.1020274	*Xestospongia muta*	24.957076	-80.460613	21213
SS.27.3.1019917	*Ircinia variabilis*	43.19794	5.363352	13103
Webster.1.C10.34.1019596	*Ianthella basta*	-9.74967	143.39925	1552
Webster.1.D4.40.1020364	*Ianthella basta*	-18.82257	147.63755	16759
Webster.1.D6.42.1020464	*Ianthella basta*	-18.82257	147.63755	15475
Webster.1.E8.56.1019871	*Ianthella basta*	-18.82257	147.63755	14296
Webster.3.A3.191.1019662	*Carteriospongia foliascens*	-16.75408	145.98733	17995
Webster.3.A4.192.1019606	*Carteriospongia foliascens*	-16.75408	145.98733	20879
Webster.3.A8.196.1020298	*Carteriospongia foliascens*	-16.75408	145.98733	15571
Webster.3.A9.197.1020023	*Carteriospongia foliascens*	-16.75408	145.98733	37317
Webster.3.B1.201.1020240	*Carteriospongia foliascens*	-16.75408	145.98733	14794
Webster.3.B8.208.1020140	*Carteriospongia foliascens*	-16.75408	145.98733	17294
Webster.3.C7.219.1019964	*Carteriospongia foliascens*	-18.81707	147.63232	15429
Webster.3.C8.220.1020034	*Carteriospongia foliascens*	-18.81707	147.63232	18105
Webster.3.D12.236.1019651	*Carteriospongia foliascens*	-18.68543	146.51218	10120
Webster.3.D4.228.1020333	*Carteriospongia foliascens*	-18.81707	147.63232	16965
Webster.3.D7.231.1020292	*Carteriospongia foliascens*	-18.68543	146.51218	18989
Webster.3.E4.240.1019504	*Carteriospongia foliascens*	-18.68543	146.51218	13654
Webster.3.E7.243.1019748	*Carteriospongia foliascens*	-18.68543	146.51218	11636

Webster.3.E8.244.1020063	*Carteriospongia foliascens*	-18.68543	146.51218	14807
Webster.3.F11.259.1020559	*Carteriospongia foliascens*	-18.5996	146.49222	18431
Webster.3.F2.250.1019808	*Carteriospongia foliascens*	-18.5996	146.49222	18595
Webster.3.F5.253.1019731	*Carteriospongia foliascens*	-18.5996	146.49222	27652
Webster.3.F9.257.1019705	*Carteriospongia foliascens*	-18.5996	146.49222	33059
Webster.3.H10.282.1020179	*Carteriospongia foliascens*	-15.3363	123.5129	26300
Webster.3.H3.275.1019734	*Carteriospongia foliascens*	-15.33242	124.243	7434
Webster.3.H5.277.1020338	*Carteriospongia foliascens*	-15.3363	123.5129	22147
Webster.3.H7.279.1019697	*Carteriospongia foliascens*	-20.64983	116.43683	10125
Webster.3.H8.280.1020167	*Carteriospongia foliascens*	-15.50517	123.60483	35589
Webster.3.H9.281.1019735	*Carteriospongia foliascens*	-15.3363	123.5129	38535
Webster.4.A6.288.1019940	*Carteriospongia foliascens*	-19.79372	149.17307	11287
Webster.4.A7.289.1019759	*Carteriospongia foliascens*	-18.6397	147.04775	17405
Webster.4.B5.299.1019876	*Ircinia*	-18.68543	146.51218	14252
Webster.4.B6.300.1019971	*Ircinia*	-18.68543	146.51218	13995
Webster.4.G7.361.1020363	*Cinachyrella alloclada*	-9.82425	150.81788	19819
Webster.4.G9.363.1020111	*Cinachyrella alloclada*	-9.82425	150.81788	15618
Webster.4.H5.371.1019922	*Cinachyrella alloclada*	-9.82805	150.82028	15305
Webster.4.H8.374.1020510	*Cinachyrella alloclada*	-9.82805	150.82028	19599
Webster.5.A1.377.1020553	*Iotrochota*	-12.484783	130.78445	4029
Webster.5.A11.387.1019740	*Halichondria phakellioides*	-12.46725	130.829517	12433
Webster.5.A12.388.1020144	*Halichondria phakellioides*	-12.46725	130.829517	9752
Webster.5.A3.379.1019907	*Iotrochota*	-12.484783	130.78445	5971
Webster.5.A4.380.1020194	*Iotrochota*	-12.484783	130.78445	5887
Webster.5.A5.381.1020416	*Spheciospongia vagabunda*	-12.418383	130.8146	4711
Webster.5.A6.382.1019604	*Spheciospongia vagabunda*	-12.418383	130.8146	12451
Webster.5.A9.385.1019972	*Halichondria phakellioides*	-12.484783	130.78445	15858
Webster.5.B5.393.1020076	*Iotrochota*	-12.46725	130.829517	8184
Webster.5.B7.395.1019832	*Spheciospongia vagabunda*	-12.46725	130.829517	7650
AF10.5.15.1181407	*Sarcotragus fasciculatus*	41.68	2.81	28259
AF10.8.15.1182052	*Sarcotragus fasciculatus*	41.68	2.81	28510
AF10.9.15.1181763	*Sarcotragus fasciculatus*	41.68	2.81	27358
IF5.3.1181746	*Sarcotragus fasciculatus*	41.38	2.2	15440
IO2.18.1181332	*Ircinia oros*	41.38	2.2	24181
IO5.25.1182429	*Ircinia oros*	41.38	2.2	13571
PO10.5.1.1181804	*Ircinia oros*	41.67	2.8	21841
TO10.10.7.1181236	*Ircinia oros*	41.72	2.94	23043
TO10.7.22.1181634	*Ircinia oros*	41.72	2.94	19014
TO10.7.7.1182167	*Ircinia oros*	41.72	2.94	17875
TO10.8.19.1181194	*Ircinia oros*	41.72	2.94	22007
TO10.9.22.1181891	*Ircinia oros*	41.72	2.94	18002
TO10.9.7.1182379	*Ircinia oros*	41.72	2.94	23760
TO11.1.22.1181309	*Ircinia oros*	41.72	2.94	17483
TV10.10.12.1181903	*Ircinia variabilis*	41.72	2.94	32022
TV10.10.7.1181679	*Ircinia variabilis*	41.72	2.94	22982

TV10.4.2.1181247	*Ircinia variabilis*	41.72	2.94	29271
TV10.8.12.1181313	*Ircinia variabilis*	41.72	2.94	37608
TV10.8.2.1181576	*Ircinia variabilis*	41.72	2.94	31932
TV10.9.12.1181059	*Ircinia variabilis*	41.72	2.94	26422
TV10.9.2.1182146	*Ircinia variabilis*	41.72	2.94	3775
TV10.9.7.1181743	*Ircinia variabilis*	41.72	2.94	25135
TV11.7.7.1181561	*Ircinia variabilis*	41.72	2.94	33668
TV11.8.12.1181970	*Ircinia variabilis*	41.72	2.94	23206
10.102009	*Ircinia strobilina*	18.40198	-87.40878	7233
100.1019549	*Xestospongia*	17.88828	-66.99812	30984
105.1019767	*Xestospongia*	17.88828	-66.99812	28264
11.1019601	*Ircinia strobilina*	18.40198	-87.40878	6191
110.1020489	*Xestospongia*	18.39177	-67.47597	24834
116.1019727	*Xestospongia*	18.39177	-67.47597	20770
12.1020554	*Ircinia strobilina*	18.40198	-87.40878	3854
120.1019581	*Xestospongia*	22.6045	-73.54653	20687
125.1020059	*Xestospongia*	24.04055	-74.53137	21614
126.1019975	*Xestospongia*	24.04055	-74.53137	23152
127.1019923	*Xestospongia*	22.10087	-73.54073	23774
128.1019745	*Xestospongia*	18.39177	-67.47597	21800
20.X.00.1.015.1020304	*Discodermia*	26.51255	-78.58482	14478
24.1020549	*Ircinia strobilina*	18.40198	-87.40878	24968
41.1019582	*Mycale laxissima*	17.95522	-67.05322	7206
47.1019927	*Mycale laxissima*	17.95522	-67.05322	13904
48.1019786	*Mycale laxissima*	17.95522	-67.05322	27172
49.1019629	*Mycale laxissima*	17.95522	-67.05322	17579
5.11.4D.1020074	*Xestospongia muta*	26.50992	-80.03365	8132
5.11.6D.1020166	*Xestospongia muta*	26.50992	-80.03365	16883
5.11.8D.1019704	*Xestospongia muta*	26.50992	-80.03365	8620
5.11.Soil.1020271	*Xestospongia muta*	26.50992	-80.03365	6124
5.29.Soil.1019966	*Xestospongia muta*	26.16817	-80.07667	13609
5.IV.91.2.003.1020531	*Axinella*	28.1595	-14.10167	5131
56.1020286	*Mycale laxissima*	17.95522	-67.05322	21234
67.1019591	*Xestospongia*	20.38228	-87.0291	16377
700.1181449	*Placospongia intermedia*	9.30683	-82.17417	3380
701.118144	*Placospongia intermedia*	9.30683	-82.17417	23844
702.118179	*Placospongia intermedia*	9.36068	-82.278	5787
712.1181913	*Aplysina fulva*	9.36068	-82.278	10431
73.1019805	*Xestospongia*	17.88828	-66.99812	26833
94.1020316	*Xestospongia*	17.88828	-66.99812	18414
95.1019987	*Plakortis*	17.88828	-66.99812	29554
99.1019716	*Plakortis*	17.88828	-66.99812	21404
ALG12.101.1020199	*Cliona viridis*	37.069333	-8.331138889	32436
ALG12.103.1020514	*Cliona celata complex*	37.069333	-8.331138889	18976
ALG12.105.1019558	*Phorbas fictitius*	37.087972	-8.342583	25877

ALG12.106.1020012	*Phorbas fictitius*	37.087972	-8.342583	16044
ALG12.110.1020285	*Cliona celata complex*	37.087972	-8.342583	25654
ALG12.111.1019802	*Cliona celata complex*	37.087972	-8.342583	21522
ALG12.112.1019618	*Cliona celata complex*	37.087972	-8.342583	26707
ALG12.114.1019900	*Cliona celata complex*	37.087972	-8.342583	21721
ALG12.115.1020456	*Cliona celata complex*	37.087972	-8.342583	17608
ALG12.95.1019784	*Phorbas fictitius*	37.069333	-8.331138889	23384
BCD12SED.1019631	Marine sediments	26.1494	-80.0971	22374
BCD13SED.1020512	Marine sediments	26.1494	-80.0971	12416
BCD21SED.1019899	Marine sediments	26.1494	-80.0971	9984
BCD22SED.1019908	Marine sediments	26.1494	-80.0971	13050
BCD23SED.1020540	Marine sediments	26.1494	-80.0971	19261
BCD31SED.1019600	Marine sediments	26.1494	-80.0971	12223
BCD32SED.1019628	Marine sediments	26.1494	-80.0971	9339
BCD33SED.1020484	Marine sediments	26.1494	-80.0971	24127
BCH12SED.1020332	Marine sediments	26.1494	-80.0971	5812
BCH13SED.1020409	Marine sediments	26.1494	-80.0971	30505
BCH21SED.1019500	Marine sediments	26.1494	-80.0971	25107
BCH32SED.1019565	Marine sediments	26.1494	-80.0971	12353
BCH33SED.1019842	Marine sediments	26.1494	-80.0971	9863
BR10B.Jan.1020118	Seawater	26.2516	-80.062	12288
BR10B.Nov.1019955	Seawater	26.2509	-80.0621	29339
BR10C.Jan.1020239	Seawater	26.2516	-80.0619	10871
BR10C.Nov.1019679	Seawater	26.251	-80.062	23631
BR14A.Jan.1020379	Seawater	26.2618	-80.0853	13317
BR7C.Jul.1020534	Seawater	26.2036	-80.0682	2086
CL22.1020317	*Cliona delitrix*	24.54912	-81.37958	9405
CL23.1019991	*Cliona delitrix*	24.54912	-81.37958	8968
CL264.1019976	*Cliona delitrix*	24.6715	-81.0506	14575
CL300.1019671	*Cliona delitrix*	25.84217	-80.10403	9866
CL416.1020174	*Cliona delitrix*	24.60903	-82.94957	15128
CL51.1019843	*Cliona delitrix*	9.3777	-82.3032	7482
Cl178.1020401	*Cliona delitrix*	16.80083	-88.07887	12771
GC.2.4.1020212	*Stylissa carteri*	20.280232	38.512573	24955
HW4A.Nov.1019983	Seawater	26.019	-80.0858	15411
HW4C.Jul.1019970	Seawater	26.0191	-80.086	14257
HW4C.Nov.1019848	Seawater	26.0193	-80.086	20420
HW9A.Jul.1020291	Seawater	26.0675	-80.0842	10947
HW9A.Nov.1019960	Seawater	26.0676	-80.0844	23100
HW9C.Jan.1019820	Seawater	26.0668	-80.0863	20729
HW9C.Jul.1020373	Seawater	26.0675	-80.0846	14323
ML2.water.gDNA.1019528	Seawater	17.955217	-67.053217	16807
ML3.water.gDNA.1019985	Seawater	17.955217	-67.053217	18363
N50.3.1.11.BC.1019585	*Amphimedon compressa*	26.15995	-80.0825	7264
P12x102.1019625	*Niphates erecta*	9.36068	-82.278	4087

P12x110.1020230	*Aiolochroia crassa*	9.36068	-82.278	21045
P12x118.1020030	*Niphates erecta*	9.36068	-82.278	4726
P12x125.1020301	*Mycale laevis*	9.35317	-82.261	20536
P12x129.1019770	*Iotrochota birotulata*	9.35317	-82.261	29989
P12x13.1020313	*Ectyoplasia ferox*	9.24133	-82.17367	21248
P12x130.1020344	*Mycale laevis*	9.35317	-82.261	13678
P12x132.1019839	*Iotrochota birotulata*	9.35317	-82.261	17110
P12x19.1019691	*Ectyoplasia ferox*	9.24133	-82.17367	14102
P12x20.1020408	*Ectyoplasia ferox*	9.24133	-82.17367	28849
P12x73.1020335	*Chondrilla caribensis*	9.35167	-82.2595	13633
P12x75.1019915	*Chondrilla caribensis*	9.35167	-82.2595	16523
P31.1020110	*Dysidea etheria*	9.30583	-82.17317	9346
SD11SED.1020180	Marine sediments	26.1673	-80.0894	18981
SD12SED.1019847	Marine sediments	26.1673	-80.0894	19898
SD21SED.1020473	Marine sediments	26.1673	-80.0894	3258
SD22SED.1020493	Marine sediments	26.1673	-80.0894	28220
SD23SED.1020164	Marine sediments	26.1673	-80.0894	26864
SD31SED.1020543	Marine sediments	26.1673	-80.0894	20905
SD33SED.1019711	Marine sediments	26.1673	-80.0894	30596
SH11SED.1019845	Marine sediments	26.1673	-80.0894	25421
SH12SED.1019810	Marine sediments	26.1673	-80.0894	24017
SH13SED.1019692	Marine sediments	26.1673	-80.0894	27032
SH1H20.1020135	Seawater	26.1673	-80.0894	26784
SH21SED.1020160	Marine sediments	26.1673	-80.0894	4675
SH23SED.1020048	Marine sediments	26.1673	-80.0894	16939
SH2H2O.1020112	Seawater	26.1673	-80.0894	29946
SH31SED.1019561	Marine sediments	26.1673	-80.0894	15235
SH32SED.1020463	Marine sediments	26.1673	-80.0894	4147
SH33SED.1020220	Marine sediments	26.1673	-80.0894	14939
SI06.133.1019903	*Lissodendoryx colombiensis*	9.36068	-82.278	22197
SI06.53.1020326	*Lissodendoryx colombiensis*	9.35167	-82.2595	29383
SI06.81.1020040	*Dysidea etheria*	9.30583	-82.17317	16990
SI06.96.1020259	*Dysidea etheria*	9.30583	-82.17317	16578
SS.11.1.1019652	*Ircinia*	-18.819402	147.649572	24749
SS.12.2.1019981	*Xestospongia testudinaria*	-18.819402	147.649572	36640
SS.25.3.1019611	*Aplysina aerophoba*	45.131314	13.666244	21580
SS.3.1.1019571	*Stelletta*	-36.37932	174.823554	11269
SS.3.2.1019835	*Stelletta*	-36.37932	174.823554	11566
SS.3.3.1019673	*Stelletta*	-36.37932	174.823554	13156
SS.5.2.1019874	*Stelletta*	-41.333	174.75	10936
SS.6.1.1020480	*Ancorina*	-41.333	174.75	10703
SS.9.1.1020557	*Cymbastela coralliophila*	-18.819402	147.649572	70634
SS.9.2.1019758	*Cymbastela coralliophila*	-18.819402	147.649572	81687
SS.9.3.1019851	*Cymbastela coralliophila*	-18.819402	147.649572	110456
SW.16.1019912	*Axinella*	59.000278	11.185	18653

SW.19.1019641	*Axinella*	58.961667	11.3275	17998
SW.227.1020120	*Myxilla*	59.043056	11.242222	7202
SW.233.1020497	*Myxilla*	59.043056	11.242222	5578
SW.234.1019718	*Myxilla*	59.043056	11.242222	4454
SW.241.1020020	*Halichondria panicea*	58.983333	11.276667	19490
SW.25.1020395	*Axinella infundibuliformis*	58.896667	11.270833	7099
SW.26.1019992	*Axinella infundibuliformis*	58.896667	11.270833	6282
SW.27.1019787	*Axinella infundibuliformis*	58.896667	11.270833	3289
SW.60.1020016	*Halichondria panicea*	58.829116	11.083156	18311
SW.H2O.4.1020393	Seawater	58.829116	11.083156	19085
SWBHSS2.1019559	Seawater	24.04055	-74.531367	28133
SWMX1.1.1020238	Seawater	20.382283	-87.0291	17491
SWMX1.2.1020168	Seawater	20.382283	-87.0291	15984
SWMX1.3.1019958	Seawater	20.382283	-87.0291	18249
SWPRBV1.1020450	Seawater	17.888283	-66.998117	29171
Webster.1.B11.23.1020420	*Ianthella basta*	-9.74967	143.39925	14375
Webster.1.C3.27.1020147	*Ianthella basta*	-9.74967	143.39925	17032
Webster.1.C7.31.1019656	*Ianthella basta*	-9.74967	143.39925	14628
Webster.1.D7.43.1019506	*Stylissa*	-18.82257	147.63755	56330
Webster.1.D8.44.1020056	*Stylissa*	-18.82257	147.63755	17300
Webster.2.D4.133.1019984	*Rhopaloeides odorabile*	-18.84478	147.64402	16399
Webster.2.E6.147.1019885	*Rhopaloeides odorabile*	-18.84478	147.64402	37608
Webster.3.A2.190.1019569	*Carteriospongia foliascens*	-16.75408	145.98733	30097
Webster.3.A6.194.1020461	*Carteriospongia foliascens*	-16.75408	145.98733	11524
Webster.3.B10.210.1019644	*Carteriospongia foliascens*	-16.75408	145.98733	17517
Webster.3.B4.204.1020234	*Carteriospongia foliascens*	-16.75408	145.98733	16399
Webster.3.B7.207.1020441	*Carteriospongia foliascens*	-16.75408	145.98733	17315
Webster.3.C9.221.1020122	*Carteriospongia foliascens*	-18.81707	147.63232	27930
Webster.3.E9.245.1019861	*Carteriospongia foliascens*	-18.68543	146.51218	41827
Webster.3.G11.271.1019913	*Carteriospongia foliascens*	-15.87005	123.66517	18262
Webster.3.G3.263.1019818	*Carteriospongia foliascens*	-16.05308	123.35887	8589
Webster.3.H2.274.1019764	*Carteriospongia foliascens*	-20.515	116.67033	9956
Webster.3.H4.276.1019944	*Carteriospongia foliascens*	-15.33242	124.243	11728
Webster.3.H6.278.1019825	*Carteriospongia foliascens*	-15.33242	125.77433	10919
Webster.4.A9.291.1020187	*Carteriospongia foliascens*	-9.75403	143.41833	16975
Webster.4.B8.302.1020437	*Luffariella variabilis*	-18.68543	146.51218	19679
Webster.4.D4.322.1020071	*Coscinoderma*	-18.59297	46.48359	6821
Webster.4.D7.325.1019936	*Coscinoderma*	-18.59297	46.48359	19289
Webster.4.D9.327.1020502	*Coscinoderma*	-18.59297	46.48359	10732
Webster.4.E3.333.1020445	*Coscinoderma*	-18.59297	46.48359	10976
Webster.4.E7.337.1020077	*Coscinoderma*	-18.59297	46.48359	19814
Webster.4.F10.352.1019826	*Rhopaloeides odorabile*	-18.81723	147.63252	7337
Webster.4.F9.351.1019995	*Rhopaloeides odorabile*	-18.81723	147.63252	11091
Webster.4.G10.364.1020027	*Cinachyrella alloclada*	-9.82425	150.81788	8952
Webster.4.G4.358.1020209	*Coelocarteria singaporensis*	-9.82425	150.81788	14438

Webster.4.G8.362.1019865	*Cinachyrella alloclada*	-9.82425	150.81788	18085
Webster.4.H6.372.1020405	*Cinachyrella alloclada*	-9.82805	150.82028	11999
Webster.5.A10.386.1019689	*Halichondria phakellioides*	-12.484783	130.78445	7154
Webster.5.B9.397.1019542	*Paratetilla*	-12.484783	130.78445	8097
Webster.5.C11.411.1020079	*Stylissa massa*	-9.82805	150.82028	10634
Webster.5.C2.402.1020087	*Stylissa massa*	-9.82425	150.81788	11305
Webster.5.C3.403.1020469	*Stylissa massa*	-9.82425	150.81788	18997
Webster.5.C4.404.1020430	*Stylissa massa*	-9.82425	150.81788	7991
Webster.5.C5.405.1020347	*Stylissa massa*	-9.82425	150.81788	7646
Webster.5.C6.406.1020060	*Stylissa massa*	-9.82425	150.81788	9538
Webster.5.C7.407.1019765	*Stylissa massa*	-9.82805	150.82028	5085
Webster.5.C9.409.1020124	*Stylissa massa*	-9.82805	150.82028	5624
Webster.5.D7.419.1019564	*Suberites diversicolour*	-12.41482	130.83212	20959
Webster.5.E4.428.1019670	Seawater	-18.82257	147.63755	20105
Webster.5.E5.429.1019889	Seawater	-18.82257	147.63755	36094
ZA.13.1020462	*Xestospongia*	-6.11738	39.16904	38208
ZA.15.1019642	*Cinachyrella*	-6.11738	39.16904	22331
ZA.16.1019749	*Cinachyrella*	-6.11738	39.16904	27082
ZA.38.1020095	*Callyspongia*	-6.149923	39.131603	4565
ZA.40.1019544	*Clathria*	-6.11738	39.16904	13842
ZA.41.1020474	*Clathria*	-6.11738	39.16904	9657
ZA.42.1019809	*Clathria*	-6.11738	39.16904	4475
ZA.46.1020417	*Cymbastela*	-6.165917	39.202641	12927
AF10.7.15.1182210	*Sarcotragus fasciculatus*	41.68	2.81	37615
AF10.7.7.1181843	*Sarcotragus fasciculatus*	41.68	2.81	29495
AF10.7.9.1181539	*Sarcotragus fasciculatus*	41.68	2.81	32729
AF10.8.9.1181531	*Sarcotragus fasciculatus*	41.68	2.81	27304
AF11.3.9.1182276	*Sarcotragus fasciculatus*	41.68	2.81	16753
AF11.8.9.1181747	*Sarcotragus fasciculatus*	41.68	2.81	27771
AW10.12.II.1182012	Seawater	41.68	2.81	4948
AW10.3.I.1181981	Seawater	41.68	2.81	14482
AW10.5.II.1182241	Seawater	41.68	2.81	19078
AW10.5.III.1182271	Seawater	41.68	2.81	13234
AW11.3.II.1181386	Seawater	41.68	2.81	3853
AW11.5.III.1181684	Seawater	41.68	2.81	8631
CCBH.1.1181350	*Crambe crambe*	41.67	2.8	22198
CCBH.3.1181704	*Crambe crambe*	41.67	2.8	25795
CCSA.1.1182079	*Crambe crambe*	41.67	2.8	20287
CCSA.3.1181521	*Crambe crambe*	41.67	2.8	18104
IF2.WIII.1181920	Seawater	41.38	2.2	28075
IO2.24.1181173	*Ircinia oros*	41.38	2.2	16675
IO2.25.1182327	*Ircinia oros*	41.38	2.2	20784
IO2.36.1182150	*Ircinia oros*	41.38	2.2	21365
IO5.18.1181378	*Ircinia oros*	41.38	2.2	21349
IO5.Wctr.1182213	Seawater	41.38	2.2	23758

IO5.Wnh.1181835	Seawater	41.38	2.2	26146
PO10.3.7.1181810	*Ircinia oros*	41.67	2.8	22245
TO10.6.22.1182060	*Ircinia oros*	41.72	2.94	20116
TO10.6.7.1181390	*Ircinia oros*	41.72	2.94	17577
TO10.8.22.1181724	*Ircinia oros*	41.72	2.94	24567
TO11.4.7.1181284	*Ircinia oros*	41.72	2.94	23726
TO11.8.7.1181052	*Ircinia oros*	41.72	2.94	18223
TV10.3.12.1182038	*Ircinia variabilis*	41.72	2.94	27182
TV10.6.12.1181765	*Ircinia variabilis*	41.72	2.94	19991
TV10.6.2.1182331	*Ircinia variabilis*	41.72	2.94	22435
TV10.6.7.1182389	*Ircinia variabilis*	41.72	2.94	23842
TV10.7.12.1181055	*Ircinia variabilis*	41.72	2.94	31194
TV11.2.2.1181754	*Ircinia variabilis*	41.72	2.94	29787
TV11.4.7.1182039	*Ircinia variabilis*	41.72	2.94	36239
TV11.6.7.1182221	*Ircinia variabilis*	41.72	2.94	32822
TV11.7.2.1181239	*Ircinia variabilis*	41.72	2.94	41814
TW10.10.II.1181942	Seawater	41.72	2.94	13163
TW10.3.I.1181457	Seawater	41.72	2.94	21455
TW11.7.I.1181050	Seawater	41.72	2.94	20266
106.1019831	*Xestospongia*	17.88828	-66.99812	25105
116.1019727	*Xestospongia*	18.39177	-67.47597	20770
52.1019713	*Mycale laxissima*	17.95522	-67.05322	1066
6.VIII.05.1.009.1019902	*Geodia*	25.69806	-79.86635	14926
69.1020426	*Xestospongia*	20.38228	-87.0291	2206
710.1181188	*Aplysina fulva*	9.36068	-82.278	18470
85.1020438	*Plakortis halichondrioides*	17.88828	-66.99812	16687
96.1019742	*Plakortis*	17.88828	-66.99812	6687
ALG12.99.1020247	*Cliona viridis*	37.069333	-8.331138889	22862
CL242.1020433	*Cliona delitrix*	26.0522	-80.09782	22806
CL43.1019930	*Cliona delitrix*	9.3777	-82.3032	11576
GC.3.4.1020086	*Aplysina aerophoba*	45.131314	13.666244	24896
P06.1019883	*Lissodendoryx colombiensis*	9.35167	-82.2595	19216
P10X38.1020293	*Haliclona vansoesti*	9.36068	-82.278	5372
P110.1019538	*Haliclona tubifera*	9.30583	-82.17317	15074
P12x105.1019499	*Aiolochroia crassa*	9.36068	-82.278	13183
P12x121.1019986	*Mycale laxissima*	9.35317	-82.261	10591
P12x126.1020142	*Mycale laevis*	9.35317	-82.261	5566
P12x127.1019894	*Iotrochota birotulata*	9.35317	-82.261	26802
P12x133.1020070	*Mycale laevis*	9.35317	-82.261	10461
P12x21.1019954	*Erylus formosus*	9.24133	-82.17367	28570
P12x51.1020198	*Haliclona tubifera*	9.30583	-82.17317	11801
P12x56.1019518	*Chalinula molitba*	9.30583	-82.17317	9235
P12x59.1019532	*Lissodendoryx colombiensis*	9.30667	-82.174	46262
P12x74.1020161	*Chondrilla caribensis*	9.35167	-82.2595	17631
SI06.27.1020539	*Tedania ignis*	9.33333	-82.25	18705

SI06.54.1019494	*Lissodendoryx colombiensis*	9.35167	-82.2595	57212
SI06.66.1020487	*Dysidea etheria*	9.30583	-82.17317	6888
SI06.9.1019630	*Xestospongia bocatorensis*	9.36068	-82.278	24174
SI06.97.1020093	*Haliclona tubifera*	9.30583	-82.17317	16845
SI06x69.1019615	*Chalinula molitba*	9.30583	-82.17317	8913
SS.27.2.1020151	*Ircinia variabilis*	43.19794	5.363352	10095
SS.29.2.1020372	*Pseudocorticium jarrei*	43.19794	5.363352	17088
SW.05.1020303	*Phakellia ventilabrum*	59.000278	11.185	13068
SW.06.1019684	*Geodia barretti*	59.000278	11.185	15182
SW.31.1020503	*Mycale lingua*	58.961667	11.3275	8728
SW.38.1020149	*Axinella infundibuliformis*	58.896667	11.270833	7415
Webster.1.C5.29.1020186	*Ianthella basta*	-9.74967	143.39925	11966
Webster.1.C9.33.1019732	*Ianthella basta*	-9.74967	143.39925	12574
Webster.1.D11.47.1020528	*Coralliophila*	-18.82257	147.63755	15305
Webster.2.A6.100.1019599	*Ircinia*	-18.56028	146.48462	12968
Webster.2.C8.125.1019896	*Rhopaloeides odorabile*	-18.84478	147.64402	12238
Webster.2.D7.136.1019776	*Rhopaloeides odorabile*	-18.84478	147.64402	30801
Webster.2.F1.154.1020479	*Rhopaloeides odorabile*	-18.84478	147.64402	23657
Webster.2.G1.166.1019779	*Rhopaloeides odorabile*	-18.84478	147.64402	22213
Webster.2.G9.174.1020452	*Rhopaloeides odorabile*	-18.84478	147.64402	17525
Webster.3.C1.213.1020348	*Carteriospongia foliascens*	-16.75408	145.98733	23126
Webster.3.C5.217.1019794	*Carteriospongia foliascens*	-18.81707	147.63232	22986
Webster.3.E6.242.1019938	*Carteriospongia foliascens*	-18.68543	146.51218	12931
Webster.3.G6.266.1020251	*Carteriospongia foliascens*	-14.17732	121.88157	9065
Webster.3.G7.267.1019856	*Carteriospongia foliascens*	-14.17705	121.8817	10648
Webster.3.H12.Negative.control.1020551	freshwater metagenome	NA	NA	5944
Webster.4.A3.285.1019579	*Carteriospongia foliascens*	-20.466	116.82817	9003
Webster.4.A5.287.1019869	*Carteriospongia foliascens*	-18.6397	147.04775	26108
Webster.4.D11.329.1020288	*Coscinoderma*	-18.59297	46.48359	8577
Webster.4.E6.336.1019575	*Coscinoderma*	-18.59297	46.48359	10108
Webster.4.G1.355.1020330	*Coelocarteria singaporensis*	-9.82425	150.81788	13389
Webster.4.G12.366.1020031	*Coelocarteria singaporensis*	-9.82805	150.82028	19214
Webster.4.H11.No.sample.1020513	freshwater metagenome	NA	NA	10212
Webster.5.D4.416.1019813	*Suberites diversicolour*	-12.41555	130.83263	15552
AF10.10.15.1181373	*Sarcotragus fasciculatus*	41.68	2.81	32038
AF10.9.7.1182028	*Sarcotragus fasciculatus*	41.68	2.81	37262
AF11.3.7.1181265	*Sarcotragus fasciculatus*	41.68	2.81	22497
IF5.27.1181406	*Sarcotragus fasciculatus*	41.38	2.2	24994
IF5.31.1181060	*Sarcotragus fasciculatus*	41.38	2.2	24315
IF5.7.1181937	*Sarcotragus fasciculatus*	41.38	2.2	19066
IO2.35.1181480	*Ircinia oros*	41.38	2.2	17746
TO10.10.19.1181285	*Ircinia oros*	41.72	2.94	29181
TO11.2.22.1181591	*Ircinia oros*	41.72	2.94	19788
TV10.11.2.1182269	*Ircinia variabilis*	41.72	2.94	26630
TV11.3.7.1182361	*Ircinia variabilis*	41.72	2.94	32450

104.1020269	*Plakortis*	17.88828	-66.99812	26355
108.1019657	*Plakortis*	17.88828	-66.99812	8949
76.1020092	*Plakortis*	17.88828	-66.99812	15162
GC.4.1.1019685	*Dysidea avara*	45.131314	13.666244	18286
Webster.1.B5.17.1020280	*Ianthella basta*	13.4513	144.65637	16761
Webster.3.B3.203.1020424	*Carteriospongia foliascens*	-16.75408	145.98733	11650
Webster.4.F8.350.1019495	*Rhopaloeides odorabile*	-18.81723	147.63252	12786
TV11.3.2.1181705	*Ircinia variabilis*	41.72	2.94	25590
5.11.9D.1019918	*Xestospongia muta*	26.50992	-80.03365	3887
GC.4.3.1020024	*Dysidea avara*	45.131314	13.666244	15823
SS.2.2.1019796	*Polymastia*	-36.37932	174.823554	8205
SS.7.3.1019590	*Xestospongia sp. UCMPWC1055*	-41.333	174.75	25352
Webster.3.F7.255.1019516	*Carteriospongia foliascens*	-18.5996	146.49222	9562
Webster.4.E10.340.1019694	*Coscinoderma*	-18.59297	46.48359	10458
AF11.1.7.1181766	*Sarcotragus fasciculatus*	41.68	2.81	36996
AF11.4.9.1181355	*Sarcotragus fasciculatus*	41.68	2.81	25719
IF5.4.1181930	*Sarcotragus fasciculatus*	41.38	2.2	26608
IO2.2.1181341	*Ircinia oros*	41.38	2.2	20397
IO2.21.1181726	*Ircinia oros*	41.38	2.2	23596
IO2.8.1181866	*Ircinia oros*	41.38	2.2	18411
TV11.1.12.1182117	*Ircinia variabilis*	41.72	2.94	35963
Webster.4.D12.330.1020385	*Coscinoderma*	-18.59297	46.48359	15594
Webster.4.E5.335.1019535	*Coscinoderma*	-18.59297	46.48359	12929
Webster.4.E8.338.1020411	*Coscinoderma*	-18.59297	46.48359	20464
Webster.4.D5.323.1019546	*Coscinoderma*	-18.59297	46.48359	14608
122.1019669	*Plakortis*	24.04055	-74.53137	26803
5.29.8I.1020184	*Xestospongia muta*	26.16817	-80.07667	13233
P12x123.1019539	*Iotrochota birotulata*	9.35317	-82.261	18125
SS.2.1.1020520	*Polymastia*	-36.37932	174.823554	9421
Webster.2.C10.127.1019906	*Rhopaloeides odorabile*	-18.84478	147.64402	13291
Webster.2.C12.129.1019806	*Rhopaloeides odorabile*	-18.84478	147.64402	11740
Webster.2.C7.124.1020296	*Rhopaloeides odorabile*	-18.84478	147.64402	24399
Webster.2.C9.126.1019979	*Rhopaloeides odorabile*	-18.84478	147.64402	10745
Webster.2.D3.132.1020457	*Rhopaloeides odorabile*	-18.84478	147.64402	12184
Webster.2.E9.150.1019527	*Rhopaloeides odorabile*	-18.84478	147.64402	12678
Webster.2.F10.163.1020475	*Rhopaloeides odorabile*	-18.84478	147.64402	11265
Webster.2.F11.164.1019681	*Rhopaloeides odorabile*	-18.84478	147.64402	9227
Webster.2.F5.158.1019833	*Rhopaloeides odorabile*	-18.84478	147.64402	18783
Webster.2.F7.160.1020229	*Rhopaloeides odorabile*	-18.84478	147.64402	29134
Webster.2.G4.169.1019852	*Rhopaloeides odorabile*	-18.84478	147.64402	23092
Webster.2.G7.172.1020355	*Rhopaloeides odorabile*	-18.84478	147.64402	27313
Webster.2.H1.178.1020495	*Rhopaloeides odorabile*	-18.84478	147.64402	10040
Webster.2.H11.188.1019926	*Carteriospongia foliascens*	-16.75408	145.98733	22646
Webster.2.H2.179.1020564	*Rhopaloeides odorabile*	-18.84478	147.64402	7720
Webster.3.A1.189.1019973	*Carteriospongia foliascens*	-16.75408	145.98733	22244

Webster.3.G8.268.1019921	*Carteriospongia foliascens*	-20.4135	116.84467	20000
Webster.4.C11.317.1020045	*Phyllospongia*	-18.68543	146.51218	8869
Webster.4.E4.334.1020244	*Coscinoderma*	-18.59297	46.48359	7293
Webster.4.F4.346.1019772	*Coscinoderma*	-18.59297	46.48359	8588
Webster.4.F5.347.1019701	*Rhopaloeides odorabile*	-18.81723	147.63252	11962
Webster.4.F6.348.1020572	*Rhopaloeides odorabile*	-18.81723	147.63252	11862
CCSA.2.1181822	*Crambe crambe*	41.67	2.8	22486
IO5.8.1181088	*Ircinia oros*	41.38	2.2	25192
2.1019675	*Mycale laxissima*	26.56295	-77.8815	12160
Webster.1.E4.52.1019857	*Ianthella basta*	-18.82257	147.63755	11039
Webster.2.D8.137.1019884	*Rhopaloeides odorabile*	-18.84478	147.64402	10452
Webster.3.A10.198.1019768	*Carteriospongia foliascens*	-16.75408	145.98733	8748
Webster.4.C8.314.1019803	*Luffariella variabilis*	-18.68543	146.51218	17773
Webster.5.D9.421.1020328	*Suberites diversicolour*	-12.41473	130.83198	10096

Table S2. Minimal inhibition concentration (MIC) of the butanone extracts of *C. violaceum* CV026-based QQ active strain against *P. aeruginosa* PAO1 (PAO1), *Bacillus subtilis* CU1050 (BS), and *Escherichia coli* GM1655 (EC)

Test extract	MIC (µg/mL)		
	EC	BS	PAO1
Pv86	500	250	500
Ac14	500	500	500
Ac4	125	250	500
Ss68	500	125	500
Pv91	250	125	500
Ss7	500	125	500
Ss38	250	250	500
Ac15	500	1000	500
Ac17	1000	1000	500
Cc27	≥2000	250	500
Cc36	≥2000	500	1000
De103	250	250	125
Ss63	250	250	500
Pv87	500	≥2000	250
Pv88	500	≥2000	1000
Pv90	250	500	500
Pv98	500	1000	250
Ampicillin	0.025	8	200

Note: Positive control- Ampicillin

Table S3. Protease activity (mean and standard deviation of duplicates) of *P. aeruginosa* in the presence of extracts of selected strains, or controls (positive control (PC): Penicillic acid, negative control (NC): methanol).

Test extract	Zone of casein hydrolysis (mm)
NC	20±0.00
Ac14	19±0.70
Ac15	20±0.00
Ac17	19±0.70
Ac4	16±1.41
Cc27	17±0.1.41
Cc36	19±0.00
De103	20±0.00
Pv86	19±0.35
Pv87	20±0.00
Pv88	20±0.70
Pv90	20±0.00
Pv91	16±0.00
Pv98	ND
Ss38	20±0.00
Ss63	20±0.70
Ss68	15±0.00
Ss7	20±0.70
PC	16±0.00

Note: Protease activity is expressed based on the diameter of the hydrolysis zone, QQ compounds will reduce the protease activity of *P. aeruginosa* PAO1, and thus, in their presence, the hydrolysis zone should be of smaller dimensions than in the negative control (<20 mm).

Table S4. List of OTUs from Sponge Microbiome project with ≥98% 16S rRNA gene identity and Score>172 against Ac4, Ac14, Ac15, Ac17, Cc27, De103, Pv86, Pv87, Pv88, Pv91, Pv98, Ss38, Ss63, Ss68 and Ss7.

Sample ID	OTU	Hit (%)	e-value	Score
Ac14	1290	100	1.00E-46	183
Ac15	1290	100	1.00E-46	183
Ac17	51717	100	1.00E-46	183
Ac4	1290	100	1.00E-46	183
Cc27	2677	100	9.00E-47	183
Cc27	20096	98.99	4.00E-45	178
Cc27	154	98.99	4.00E-45	178
De103	19426	100	4.00E-46	182
De103	4508	100	4.00E-46	182
De103	4507	100	4.00E-46	182
De103	152535	100	1.00E-45	180
De103	1438	100	1.00E-45	180
De103	745	100	1.00E-45	180
De103	548	100	1.00E-45	180
De103	85134	100	5.00E-45	178
De103	51421	98.99	5.00E-45	178
De103	21363	98.99	5.00E-45	178
De103	19425	98.99	5.00E-45	178
De103	19413	98.99	5.00E-45	178
De103	13939	98.99	5.00E-45	178
De103	12124	98.99	5.00E-45	178
De103	7321	98.99	5.00E-45	178
De103	3825	98.99	5.00E-45	178
De103	3793	98.99	5.00E-45	178
De103	1808	98.99	5.00E-45	178
De103	152322	98.99	2.00E-44	176
De103	85139	98.98	2.00E-44	176
De103	51425	100	2.00E-44	176
De103	51381	100	2.00E-44	176
De103	51311	98.98	2.00E-44	176
De103	35499	100	2.00E-44	176
De103	13978	98.98	2.00E-44	176
De103	12127	98.98	2.00E-44	176
De103	154451	98.97	6.00E-44	174
De103	152514	98.97	6.00E-44	174
De103	152388	98.97	6.00E-44	174
De103	152344	98.97	6.00E-44	174
De103	152165	98.97	6.00E-44	174
De103	152105	100	6.00E-44	174
De103	151937	98.97	6.00E-44	174

De103	151868	98.97	6.00E-44	174
De103	151848	98.97	6.00E-44	174
De103	151818	98.97	6.00E-44	174
De103	85140	98.97	6.00E-44	174
De103	85058	98.97	6.00E-44	174
De103	85031	98.97	6.00E-44	174
De103	85003	98.97	6.00E-44	174
De103	84983	98.97	6.00E-44	174
De103	84280	98.97	6.00E-44	174
De103	84241	98.97	6.00E-44	174
De103	57730	98.97	6.00E-44	174
De103	57721	98.97	6.00E-44	174
De103	57715	98.97	6.00E-44	174
De103	57711	98.97	6.00E-44	174
De103	57701	98.97	6.00E-44	174
De103	57700	98.97	6.00E-44	174
De103	57678	98.97	6.00E-44	174
De103	57676	98.97	6.00E-44	174
De103	51433	98.97	6.00E-44	174
De103	51430	98.97	6.00E-44	174
De103	51426	98.97	6.00E-44	174
De103	51412	98.97	6.00E-44	174
De103	51383	98.97	6.00E-44	174
De103	51379	98.97	6.00E-44	174
De103	51378	98.97	6.00E-44	174
De103	51367	98.97	6.00E-44	174
De103	51363	98.97	6.00E-44	174
De103	51318	98.97	6.00E-44	174
De103	35550	98.97	6.00E-44	174
De103	35542	98.97	6.00E-44	174
De103	35537	98.97	6.00E-44	174
De103	35535	98.97	6.00E-44	174
De103	35533	98.97	6.00E-44	174
De103	35532	98.97	6.00E-44	174
De103	35526	98.97	6.00E-44	174
De103	35512	98.97	6.00E-44	174
De103	35510	98.97	6.00E-44	174
De103	35507	98.97	6.00E-44	174
De103	35497	98.97	6.00E-44	174
De103	35496	98.97	6.00E-44	174
De103	35490	98.97	6.00E-44	174
De103	21408	98.97	6.00E-44	174
De103	21407	98.97	6.00E-44	174
De103	21400	98.97	6.00E-44	174
De103	21399	98.97	6.00E-44	174

De103	21398	98.97	6.00E-44	174
De103	21397	98.97	6.00E-44	174
De103	21394	98.97	6.00E-44	174
De103	21374	98.97	6.00E-44	174
De103	21368	98.97	6.00E-44	174
De103	21367	98.97	6.00E-44	174
De103	21364	98.97	6.00E-44	174
De103	21362	98.97	6.00E-44	174
De103	19427	98.97	6.00E-44	174
De103	19424	98.97	6.00E-44	174
De103	19423	98.97	6.00E-44	174
De103	19422	98.97	6.00E-44	174
De103	19421	98.97	6.00E-44	174
De103	19419	98.97	6.00E-44	174
De103	19386	98.97	6.00E-44	174
De103	19384	98.97	6.00E-44	174
De103	19383	98.97	6.00E-44	174
De103	13980	98.97	6.00E-44	174
De103	13975	98.97	6.00E-44	174
De103	13942	98.97	6.00E-44	174
De103	10494	98.97	6.00E-44	174
De103	8019	98.97	6.00E-44	174
De103	6738	98.97	6.00E-44	174
De103	6737	98.97	6.00E-44	174
De103	2432	98.97	6.00E-44	174
De103	1739	98.97	6.00E-44	174
De103	85162	98.96	2.00E-43	172
Pv86	1296	100	1.00E-46	183
Pv86	207628	98.99	5.00E-45	178
Pv86	207515	98.99	5.00E-45	178
Pv86	207359	98.99	5.00E-45	178
Pv86	138931	98.99	5.00E-45	178
Pv86	138901	98.99	5.00E-45	178
Pv86	138892	98.99	5.00E-45	178
Pv86	34154	98.99	5.00E-45	178
Pv86	20655	98.99	5.00E-45	178
Pv86	3160	98.99	5.00E-45	178
Pv87	4185	100	2.00E-46	183
Pv87	6697	98.99	9.00E-45	178
Pv87	1013	98.99	9.00E-45	178
Pv87	119214	98.97	1.00E-43	174
Pv88	2724	100	1.00E-46	183
Pv91	143340	100	1.00E-46	183
Pv98	230	100	4.00E-46	182
Pv98	3451	98.98	2.00E-44	176

Pv98	47167	98.98	7.00E-44	174
Ss38	160513	98.99	5.00E-45	178
Ss63	20072	100	1.00E-46	183
Ss63	75866	98.99	5.00E-45	178
Ss63	75850	98.99	5.00E-45	178
Ss63	18624	98.99	5.00E-45	178
Ss63	15317	98.99	5.00E-45	178
Ss68	7598	100	1.00E-46	183
Ss68	4555	98.99	5.00E-45	178
Ss7	26192	100	1.00E-46	183
Ss7	1930	98.97	6.00E-44	174

Table S5A. Selected features with their corresponding mzmed, rtmed, adducts, METLIN MS/MS match and AntiMarin database match for extract from isolate Cc27.

mzmed	rtmed	adducts	METLIN (MS/MS)	AntiMarin
359.1738	3.78	[M+Na]+ 336.184	n	n
280.1107	4.55	[M+K]+ 241.147	n	n
358.2389	5.64		n	n
339.1602	6.57		**1**	**1**
608.3939	7.70		n	**2**
284.1644	7.92	[M+H]+ 283.158	n	n
267.1153	8.98	[M+Na]+ 244.125	n	n
247.1482	9.75	[M+H]+ 246.142	n	n
343.2394	10.80	[M+Na]+320.239 [M+H-COCH2]+ 384.236	n	n
785.5158	13.00	[M+H+NH3]+ 767.482	n	n
468.3917	27.80	[M+Na+NH3]+ 428.379 [M+H+NH3]+ 450.361	n	**3**

Table S5B. Selected features with their corresponding mzmed, rtmed, adducts, METLIN MS/MS match and AntiMarin database match for extract from isolate Ss68.

mzmed	rtmed	adducts	METLIN (MS/MS)	AntiMarin
256.18	8.65		n	n
489.257	9.03	[2M+H]+ 244.125	n	n
316.171	9.42	[M+H]+ 315.158 [M+H-CH4]+ 331.207	n	n
389.257	11.2	[M+Na]+ 366.267	20991	
377.203	12	[M+H]+ 376.197	n	n
421.229	12.2	[M+H]+ 420.217	n	n

741.489	12.9	[M+K+NH3]+ 685.49<br>[M+H+NH3]+ 723.455	n	n
313.204	13.7	[M+H-C4H8]+ 368.259	44851	n
412.327	15.1	[M+Na+NH3]+ 372.292	n	
412.327	17	[M+K]+ 373.358	n	n
327.158	22.1		18061	n

Table S5C. Selected features with their corresponding mzmed, rtmed, adducts, METLIN MS/MS match and AntiMarin database match for extract from isolate Pv86.

mzmed	rtmed	adducts	METLIN (MS/MS)	AntiMarin
350.1552	8.29	[M+H]+ 349.148	n	4
334.1597	11.13	[M+K]+ 295.198	n	n
471.1864	12.01	[3M+H+Na]2+ 306.136	22979	
635.2739	12.08	[2M+Na]+ 306.136	n	5
460.3006	12.37	[M+H+NH3]+ 442.267	45418	
504.3278	12.51	[M+H+NH3]+ 486.292	n	6
456.2097	25.90		y	
412.3277	28.12		68586	

Table S5D. Selected features with their corresponding mzmed, rtmed, adducts, METLIN MS/MS match and AntiMarin database match for extract from isolate Pv91.

mzmed	rtmed	adducts	METLIN (MS/MS)	AntiMarin
426.2081	5.58	[M+K]+ 387.245	n	**7**
654.2737	7.42	[M+H]+ 653.267	n	n
688.2402	9.22		n	n
697.4609	14.14	[M+K+NH3]+ 641.459	n	**8**
812.5125	14.34	[M+Na]+ 789.516<br>[M+H-H20]+ 829.521	n	n
418.2856	14.79	[M+Na]+ 395.305	n	n
449.2575	17.17	[M+Na]+ 426.27	n	n
982.4649	18.25	[M+H+NH3]+ 964.431	n	**9**

546.3724	19.27	[M+H-CO]+ 573.37 [M+H-C2H4]+ 573.37	n	n
537.3501	19.46	[M+H]+ 536.343	n	n
399.2431	20.03		n	n
560.3876	20.23	[M+K]+ 521.439 [M+H]+ 559.385	n	n
439.3112	21		n	n
689.566	23.72	[M+Na]+ 666.585 [M+H]+ 688.559	n	n
689.5652	24.5	[2M+Na+K-H]+ 314.299 [M+H-NH3]+ 705.578	n	
542.4158	24.86	[M+K]+ 503.463 [M+Na]+ 519.415	n	n
371.1064	26.52	[M+2K]2+ 664.267 [M+Na+K]2+ 680.246	n	n

1.2 Supplementary Figures

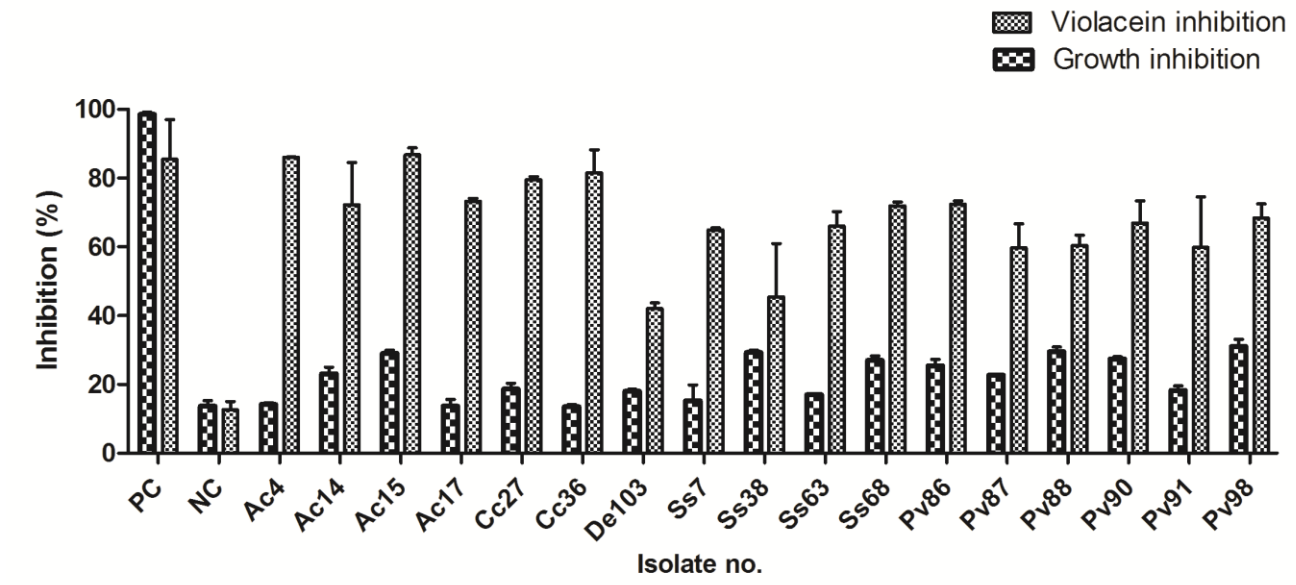

Figure S1 *C. violaceum* CV026 violacein and growth inhibition (%) in presence of extracts from 17 different isolates, Penicillic acid (0.025 mg/mL) and ampicillin (3 µg/mL) were used as the positive controls for violacein and growth inhibition assays respectively. Inhibition of violacein production or growth was calculated as percentage inhibition compared to the inhibition by the negative control (methanol).

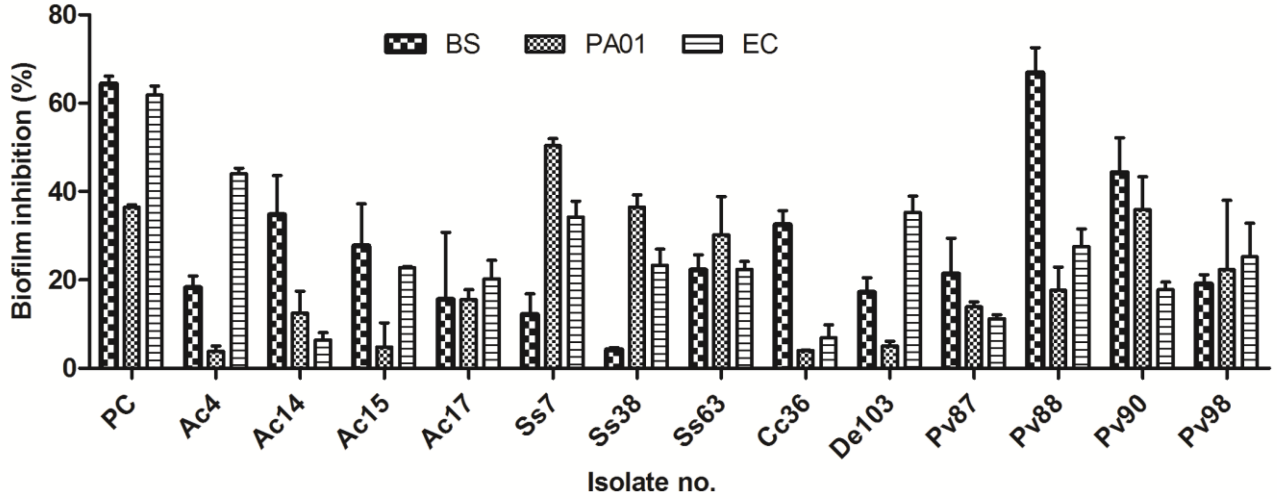

Figure S2. Inhibition of biofilm formation (%) in *P.aeruginosa* PAO1, *Bacillus substilis* and *E. coli* by extracts from isolates deriving from *Crella cyathophora* (Cc), *Diacarnus erythraenus* (De), *Pione vastifica* (Pv), *Amphimedon chloros* (Ac), *Sarcotragus sp.*(Ss). Streptomycin was used as positive control (PC) for PAO1 and BS, whereas penicillic acid was used for EC. Biofilm inhibition was calculated as percentage inhibition compared to the inhibition by the negative control (methanol). Note that some activities may have resulted from growth inhibition. Specifically extracts Ss68, Pv91 and Ss7 showed growth inhibition against BS and extract Ac4 inhibited growth of EC (see Table S2 for details on MIC).

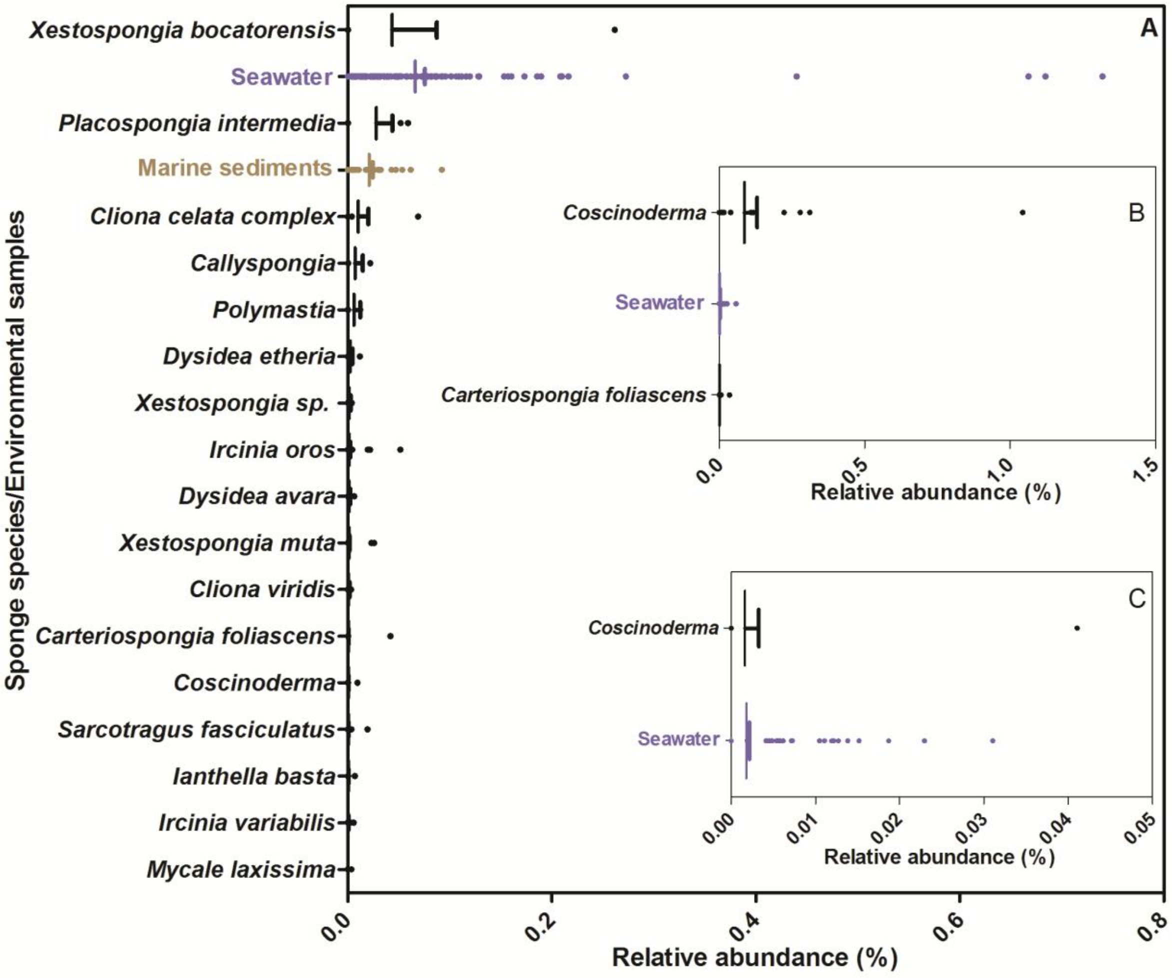

Figure S3. Relative abundance of OTUs from the SMP with ≥98% identity to 16S rRNA sequences from **A.** Information relative to OTUs closely affiliated to isolate Pv87 **B**. Information relative to OTUs closely affiliated with isolates Pv88. **C**. Information relative to OTUs closely affiliated with isolates Ss38. Vertical bar represents the mean, the hinge represents SEM (standard error mean), and dots represent outliner values beyond mean.

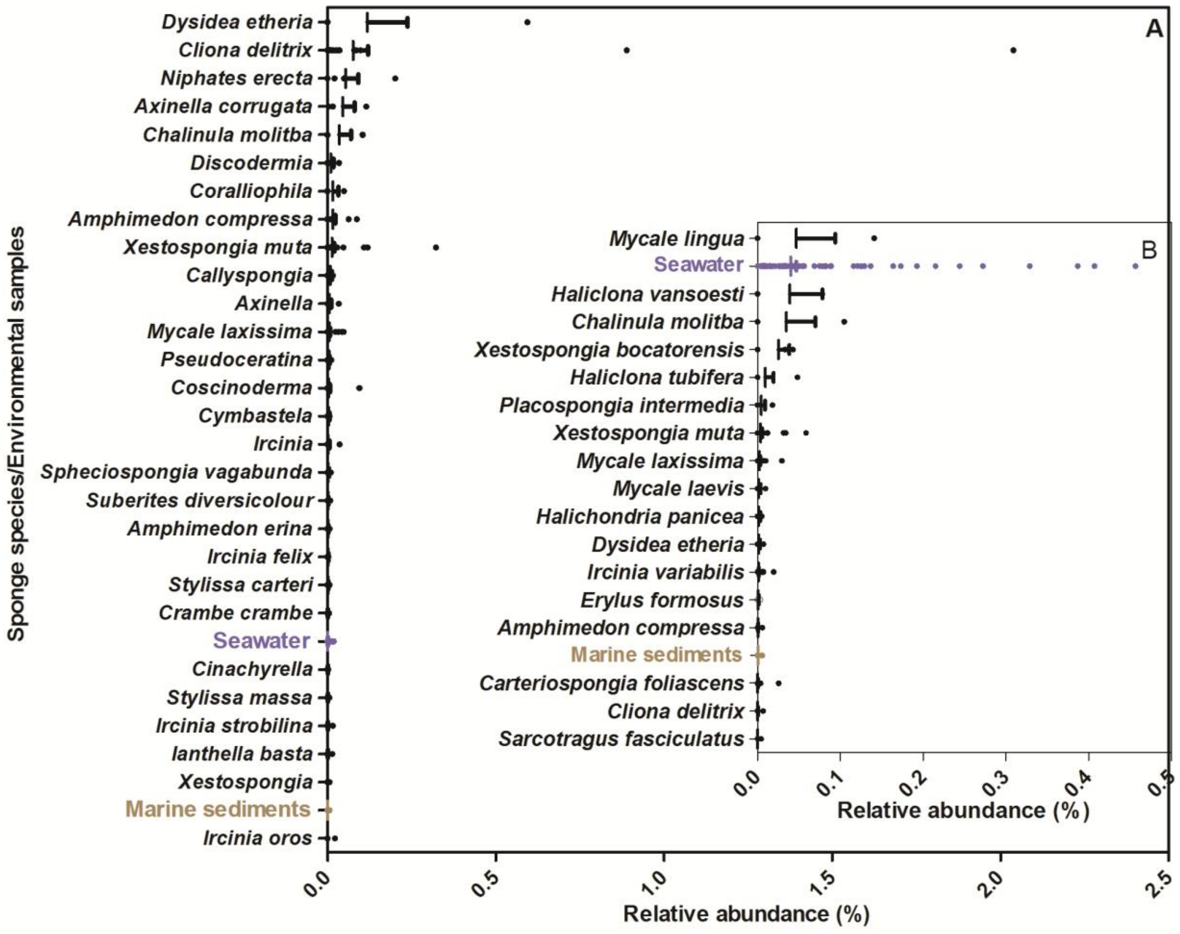

Figure S4. Relative abundance of OTUs from the SMP with ≥98% identity to 16S rRNA sequences from **A.** Information relative to OTUs closely affiliated to isolate Ac4,14,15 **B**. Information relative to OTUs closely affiliated with isolates Ss63. Vertical bar represents the mean, the hinge represents SEM (standard error mean), and dots represent outliner values beyond mean.

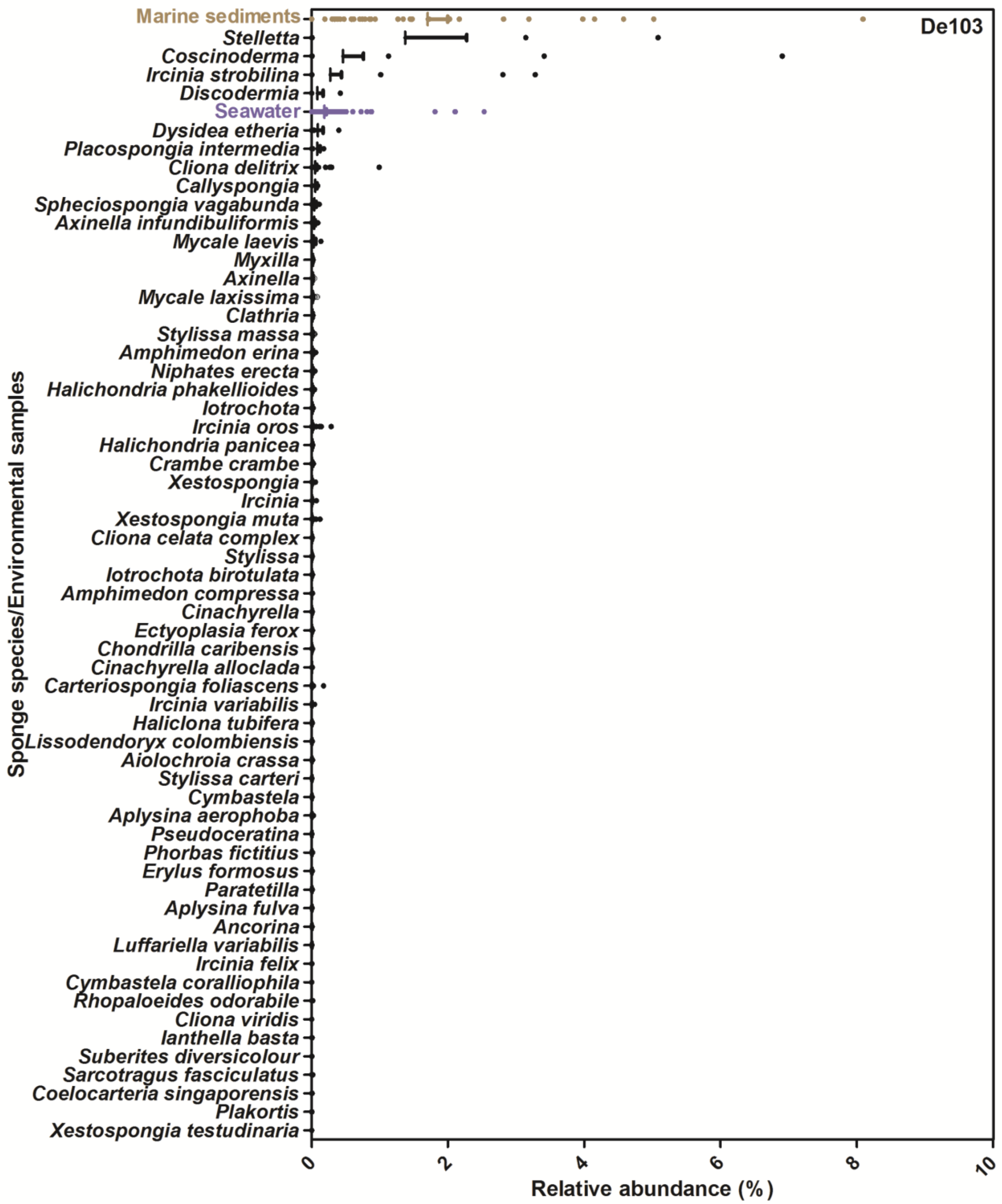

Figure S5. Relative abundance of OTUs from the SMP with ≥98% identity to 16S rRNA sequences from information relative to OTUs closely affiliated to isolate De103. Vertical bar represents the mean, the hinge represents SEM (standard error mean), and dots represent outliner values beyond mean.

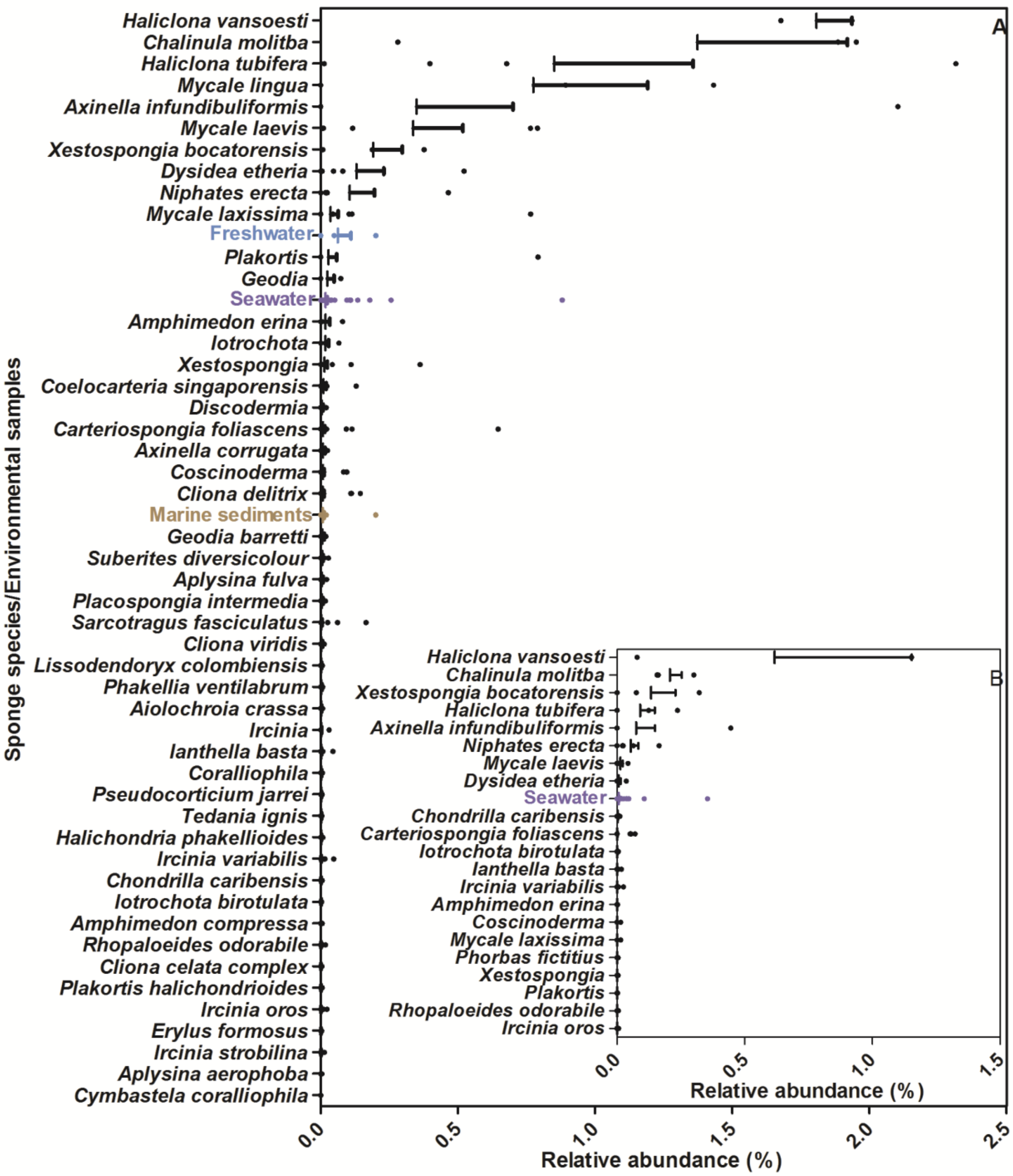

Figure S6. Relative abundance of OTUs from the SMP with ≥98% identity to 16S rRNA sequences from **A**. Information relative to OTUs closely affiliated to isolate Pv98 **B**. Information relative to OTUs closely affiliated with isolates Ss7. Vertical bar represents the mean, the hinge represents SEM (standard error mean), and dots represent outliner values beyond mean.

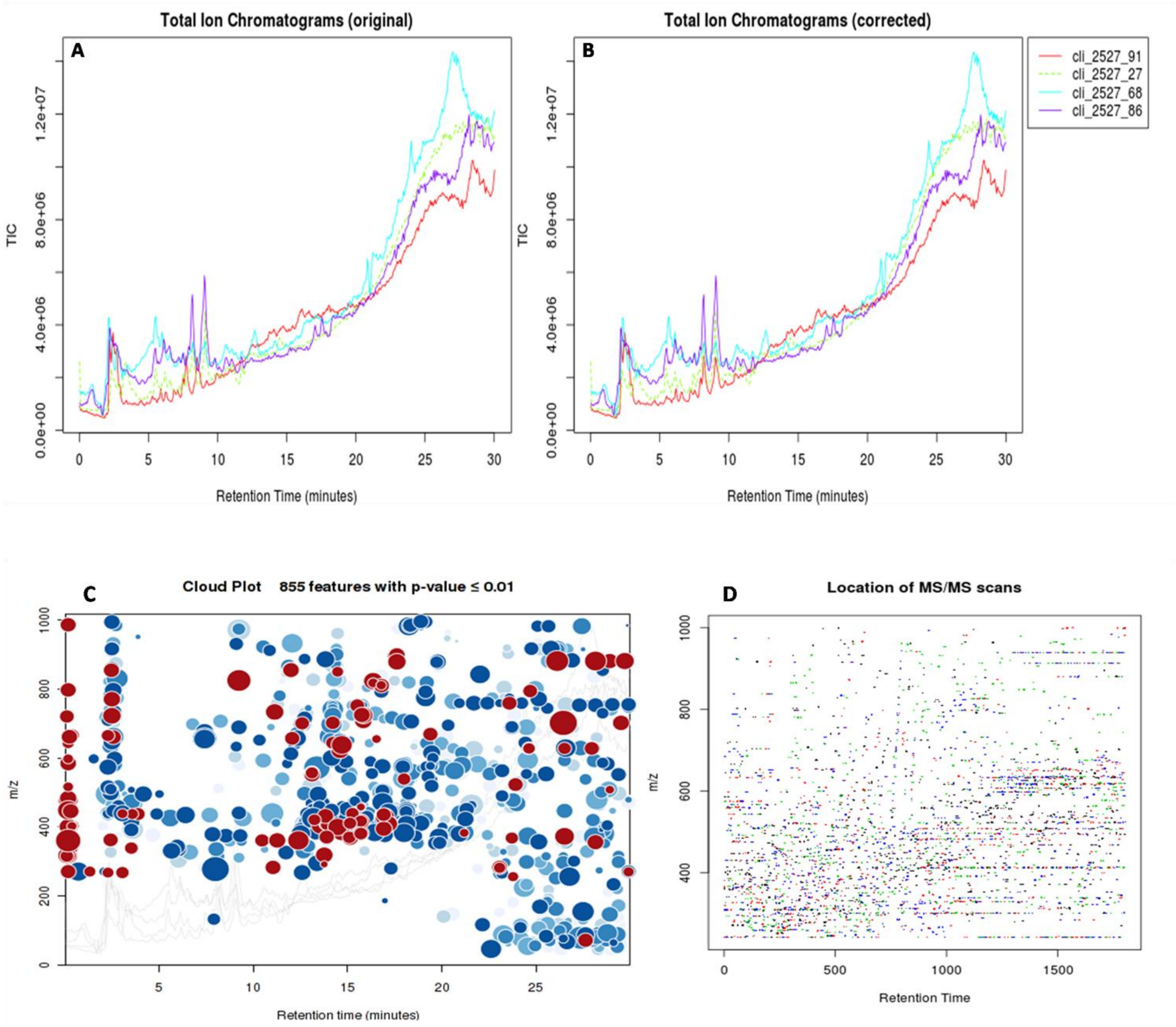

Figure S7.The overlay of total ion chromatograms before (**A**) and after (**B**) retention time correction, Cloud plots with 855 features with p-value of ≤0.001 (**C**) and location of MS/MS scans (**D**).

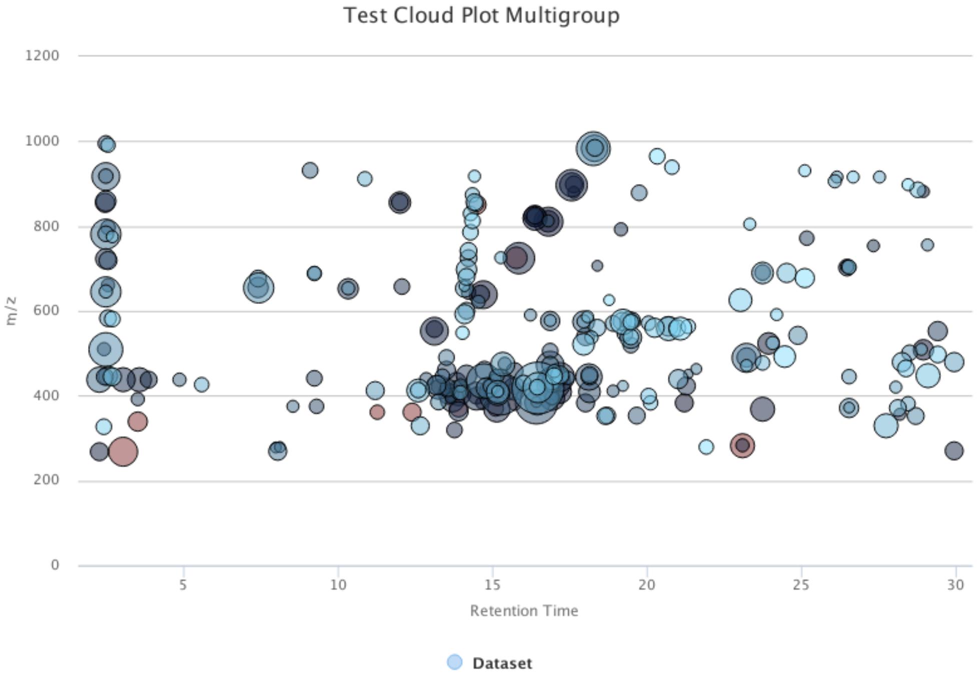

Figure S8. Interactive cloud plot analysis for the dataset containing compounds from extracts deriving from the four selected isolates (Cc27, Pv86, Pv91, and Ss68) for metabolomic profiling.
